# The catalytic and structural basis of archaeal glycerophospholipid biosynthesis

**DOI:** 10.1007/s00792-022-01277-w

**Published:** 2022-08-17

**Authors:** Niels A. W. de Kok, Arnold J. M. Driessen

**Affiliations:** Department of Molecular Microbiology, Groningen Biomolecular Sciences and Biotechnology Institute, University of Groningen, 9747AG Groningen, The Netherlands

**Keywords:** Archaea, Crystal structure, Glycerophospholipids, Lipid-divide, Lipid metabolism

## Abstract

Archaeal glycerophospholipids are the main constituents of the cytoplasmic membrane in the archaeal domain of life and fundamentally differ in chemical composition compared to bacterial phospholipids. They consist of isoprenyl chains ether-bonded to glycerol-1-phosphate. In contrast, bacterial glycerophospholipids are composed of fatty acyl chains ester-bonded to glycerol-3-phosphate. This largely domain-distinguishing feature has been termed the “lipid-divide”. The chemical composition of archaeal membranes contributes to the ability of archaea to survive and thrive in extreme environments. However, ether-bonded glycerophospholipids are not only limited to extremophiles and found also in mesophilic archaea. Resolving the structural basis of glycerophospholipid biosynthesis is a key objective to provide insights in the early evolution of membrane formation and to deepen our understanding of the molecular basis of extremophilicity. Many of the glycerophospholipid enzymes are either integral membrane proteins or membrane-associated, and hence are intrinsically difficult to study structurally. However, in recent years, the crystal structures of several key enzymes have been solved, while unresolved enzymatic steps in the archaeal glycerophospholipid biosynthetic pathway have been clarified providing further insights in the lipid-divide and the evolution of early life.

## Introduction

**Fig. 1:**
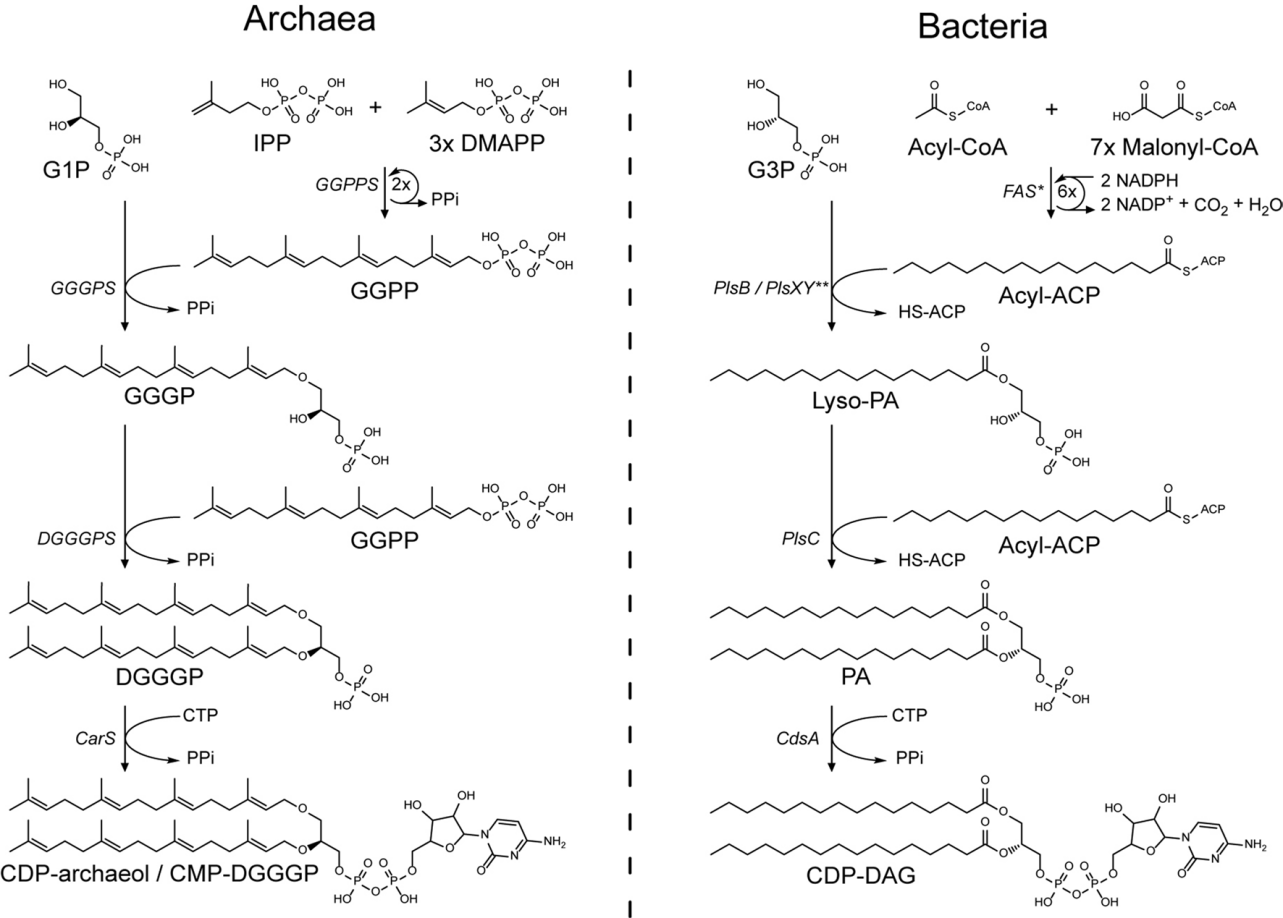
The biosynthesis pathway of the phospholipid core up to and including phospholipid headgroup activation. *In the initial fatty-acid biosynthesis reaction, both substrates are transesterified from CoA to ACP. This also occurs during each malonyl–CoA addition cycle, but these ACP groups are regenerated and therefore not shown in this step. **The PlsB of some organisms accepts acyl-CoA as well and the PlsXY pathway steps and acyl-phosphate intermediate are omitted for clarity

The cell membrane is an essential part of life. Membranes are required for cellular compartmentalization, allowing for specialized reaction compartments and for maintenance of chemical gradients across the membrane; supporting processes such as transport, ATP synthesis and motility. On the basis of ribosomal RNA sequences, in 1977, Carl Woese proposed a phylogenetic tree of life containing 3 domains: the Eukarya, Bacteria and Archaea (Woese and Fox 1977). One of the fundamental features that distinguishes Archaea from Bacteria and Eukarya is the difference in the structure of their phospholipids, the main constituent of cell membranes. Members of the domains of Bacteria and Eukarya generally synthesize phospholipids containing fatty acids esterified to glycerol-3-phosphate, whereas Archaea synthesize phospholipids containing isoprene moieties connected to glycerol-1-phosphate via ether bonds; these fundamental differences between archaeal lipids and those found in Bacteria and Eukarya are referred to as the “lipid-divide” (Koga 2011, 2014; Lombard et al. 2012a; Villanueva et al. 2017). While the nature of the hydrophobic radyl groups, the connection to the glycerol backbone, and the chirality of that backbone is markedly different; the core of the phospholipid biosynthesis pathways of Archaea and Bacteria run in parallel with a remarkably similar overall organization. However, the enzymatic reactions for the formation of the ether bonds versus the ester bonds require rather different enzymes and mechanisms (Fig. 1). This is in stark contrast with the diversification pathways of the phospholipid polar headgroups, which, for most modifications, are strikingly similar and involve similar enzymes.

Comparative studies on the structures of enzymes responsible for membrane lipid biosynthesis can provide insights in the molecular basis for the core differences between bacterial and archaeal lipids. Furthermore, these studies may deepen our understanding of the evolution of early life and what early life might have looked like. Typically, over long evolutionary periods, DNA sequences can be altered to a great extent by mutations, ultimately leading to low primary amino acid sequence homology. In the phospholipid biosynthetic enzymes, homology is primarily found in critical structural elements such as particular folds or substrate and co-factor-binding sites. In particular sequences pertaining to hydrophobic features, which are often involved in lipid binding, amino acid identities tend to diverge more over time, while amino acid properties are often more conserved. Thus, features could still be structurally conserved in the tertiary structure and be apparent in protein hydropathy profiles and crystal structures (Lolkema and Slotboom 1998a, b).

This review discusses the enzymatic basis of archaeal phospholipid biosynthesis, combining information from various works about the biochemical characteristics and structures of archaeal lipid biosynthesis enzymes to give an overview of the current state of knowledge. The combined knowledge of these fields revealed new insights which have implications for the continued characterization of the archaeal lipid biosynthesis pathway, and in extension, on the lipid-divide and evolution of early life.

### Glycerophosphate lipid core biosynthesis

#### Mevalonate (MVA) pathway

Isoprenoids constitute a very diverse group of naturally occurring compounds. Several important isoprenoid compounds include, for example, carotenoids, steroids, dolichols, plant terpenes and various prenylated compound groups such as archaeal phospholipids, quinones, chlorophylls and prenylated proteins. The essential building blocks of isoprenoids are isopentenyl pyrophosphate (IPP) and dimethylallyl pyrophosphate (DMAPP). Two non-homologous and unrelated pathways are known to exist for the synthesis of these compounds: the mevalonate (MVA) pathway and the unrelated non-mevalonate, MEP/DOXP pathway (Lombard and Moreira 2011). Additionally, to date, three alternate MVA pathways were discovered in archaea that are related to the canonical eukaryotic MVA pathway (VanNice et al. 2014; Vinokur et al. 2014; Hayakawa et al. 2018). Even though there is no clear domain-related distribution of these pathways, Eukarya and members of the Sulfolobales tend to use the canonical MVA pathway, while most other Archaea tend to use one of the three alternate MVA pathways. Bacteria generally synthesize isoprenoids through the MEP/DOXP pathway (Lombard and Moreira 2011). One of the three alternate MVA pathways found in archaea is the common “archaeal MVA pathway” named after its probable conservation in most archaea (Hayakawa et al. 2018; Yoshida et al. 2020). Archaea of the order Halobacteriales (and some members of the Chloroflexi bacteria) use the “haloarchaeal-type MVA pathway” (Dellas et al. 2013; VanNice et al. 2014), while members of the Thermoplasmatales use the “Thermoplasma-type MVA pathway” (Azami et al. 2014; Vinokur et al. 2014, 2016).

**Fig. 2:**
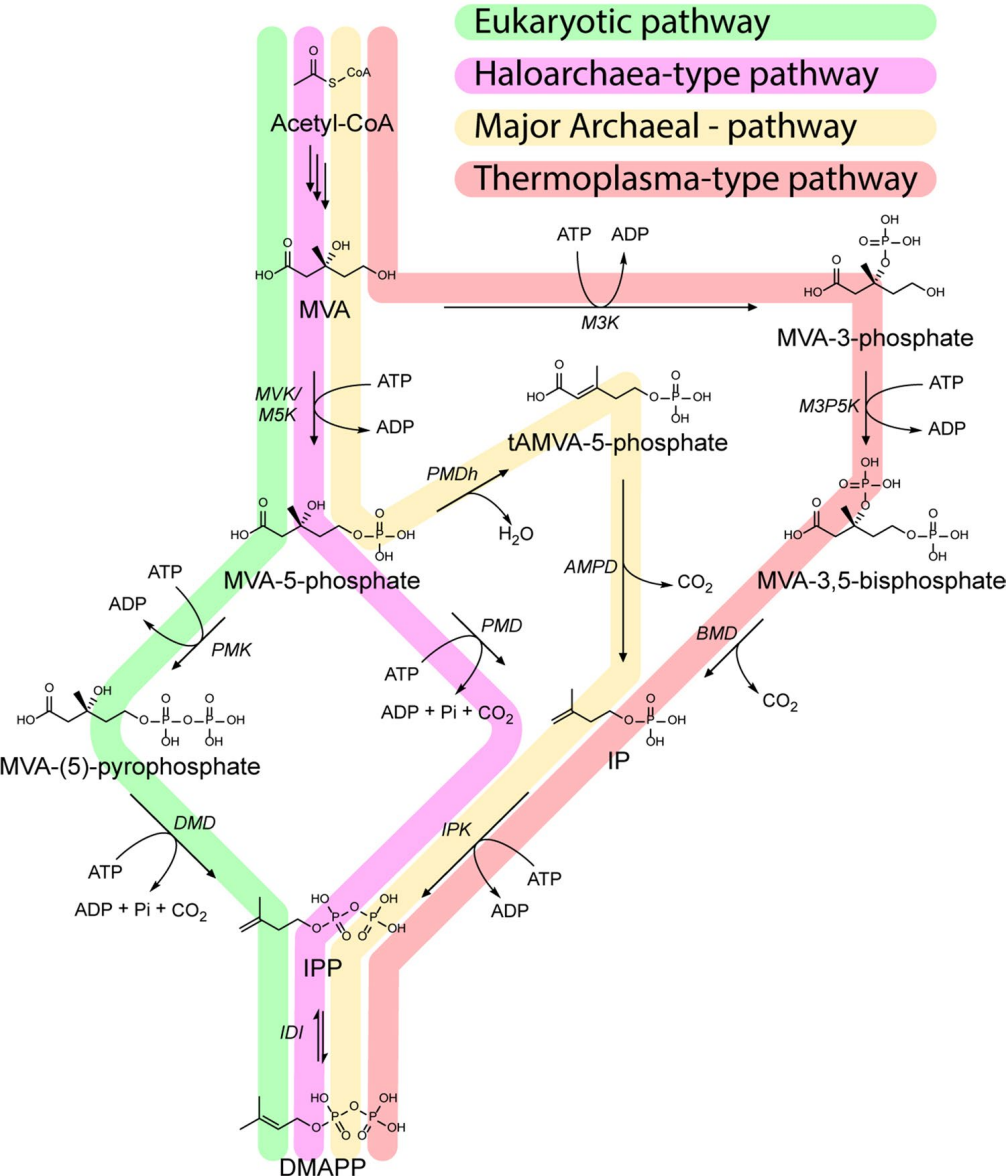
A schematic representation of the four known MVA pathways. The names of the enzymes are italicized. The pathways are marked based on their distribution. MVK, mevalonate kinase; M5K, mevalonate-5-kinase; PMK, phosphomevalonate kinase; DMD, diphosphomevalonate kinase; PMD, phosphomevalonate decarboxylase; PMDh, phosphomevalonate dehydratase; AMPD, anhydromevalonate phosphate decarboxylase; M3K, mevalonate-3 kinase; M3P5K, mevalonate-3-phosphate-5 kinase; BMD, bisphosphomevalonate decarboxylase; IDI, isopentenyl pyrophosphate:dimethylallyl pyrophosphate isomerase

The upper MVA pathway is shared between all organisms employing a MVA pathway and is responsible for the conversion of acetyl-CoA to MVA through 3 enzymatic reactions. The differences between the canonical eukaryotic- and alternate archaeal-type pathway variants are found in the later steps in the MVA pathway (Fig. 2). The canonical eukaryotic MVA pathway converts MVA into IPP in 3 steps: First, MVA is phosphorylated to mevalonate-5-phosphate (MVA-5-P) which is phosphorylated a second time to yield mevalonate-(5)-pyrophosphate (MVA-5-PP) prior to decarboxylation to form IPP. In the haloarchaeal-type MVA pathway, the second phosphorylation step and decarboxylation are swapped compared to the eukaryotic pathway: MVA is converted into IPP through MVA-5-P and isopentenyl phosphate (IP), a key intermediate for the archaeal pathway variants (Dellas et al. 2013; VanNice et al. 2014). The *Thermoplasma*-type MVA pathway converts MVA into IPP using 4 steps through the mevalonate-3-phosphate (MVA-3-P), mevalonate-3,5-biphosphate (MVA-3,5-PP) and IP intermediaries into IPP (Azami et al. 2014; Vinokur et al. 2014, 2016). The “archaeal” MVA pathway is similar to the haloarchaeal-type pathway, but employs an extra intermediate. As with the canonical eukaryotic- and haloarchaeal-type MVA pathways, MVA is phosphorylated to yield MVA-5-P. MVA-5-P is dehydrated to form trans-anhydroMVA-5-P (tAMVA-5-P) which is subsequently decarboxylated to IP and phosphorylated to form IPP (Hayakawa et al. 2018; Yoshida et al. 2020; Watanabe et al. 2021). The last step of the lower MVA pathway is shared by all MVA pathway variants and involves the formation of DMAPP through the reversible isomerization of IPP.

#### Geranylgeranyl pyrophosphate synthesis

Isoprenyl pyrophosphate chains are formed by members of the isoprenyl pyrophosphate synthase (IPPS) family belonging to the prenyltransferase superfamily. Various IPPS are capable of synthesizing different chain lengths which are then used as precursors for terpene-based molecules such as quinones, carotenoids, steroids, hopanoids, dolichols, prenylated proteins or for archaeal phospholipid biosynthesis. Archaeal diether phospholipid biosynthesis typically requires C_20_ and/or C_25_ isoprene chains which are synthesized by all-trans*,* head-to-tail, short-chain IPPS; or more specifically geranylgeranyl pyrophosphate synthase (GGPPS) and geranylfarnesyl pyrophosphate synthases (GFPPS), respectively (Fig. 1). While not used for glycerophospholipid biosynthesis in archaea, hexaprenyl pyrophosphate (HexPPS) and heptaprenyl pyrophosphate synthases (HepPPS) are also members of the isoprenyl pyrophosphate synthase family and are required for the biosynthesis of respiratory quinones (Hemmi et al. 2002; Sun et al. 2005). The mechanism of action of these enzymes is conserved throughout the all-*trans-*type IPPS family and follows a general “ionization–addition–elimination” mechanism (Lu et al. 2009). First, DMAPP is ionized, losing its pyrophosphate group to form a carbocation allowing it to act as a prenyl donor. IPP then performs a nucleophilic attack with C-4 on the C-1 of the dimethylallyl carbocation (“tail-to-head”) forming a carbon–carbon bond. This is followed by the elimination of H^+^ from C-2 of the IPP moiety, forming the typical isoprenyl *trans*-double bond, resulting in the geranyl pyrophosphate (GPP, C_10_) product (Smart and Pinsky 1977; Poulter and Satterwhite 1977; Poulter et al. 1978; Tarshis et al. 1996; Ohnuma et al. 1996b). In archaea, this reaction is iteratively repeated by GGPPS, consuming IPP, forming farnesyl pyrophosphate (FPP, C_15_); and is repeated again to form geranylgeranyl pyrophosphate (GGPP, C_20_) which can be used for the biosynthesis of archaeal phospholipids. Additionally, in some archaea, this iterative elongation reaction is repeated once more to form geranylfarnesyl pyrophosphate (GFPP, C_25_) by GFPPS for the synthesis of C_25_ archaeal lipids (Tachibana et al. 2000).

**Fig. 3:**
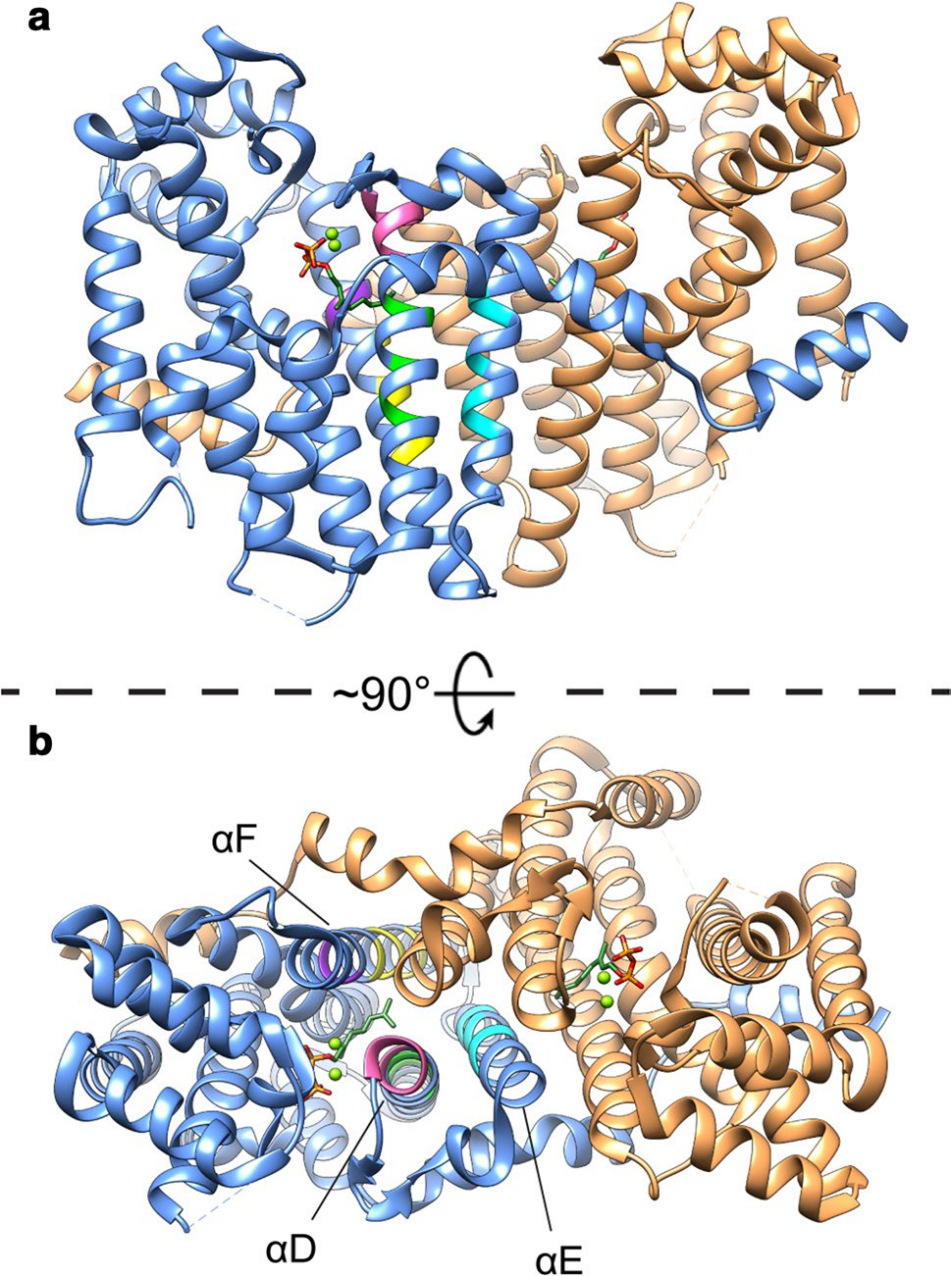
Side view (**a**) and top-down view (**b**) of the Saccharomyces cerevisiae GGPPS crystal structure with bound GPP (PDB: 2E8X, (Guo et al. 2007)). Both peptide chains of the crystal structure dimer are shown (Sandy brown for chain A and Cornflower blue for chain B). The FARM motif is highlighted in pink and the G(Q/E) motif in purple. The helices that form the product elongation cavity are annotated. Putative limiter residues based on the “three-floor” model are highlighted in yellow, green and cyan. Mg2 + ions are shown as green spheres

Remarkably, with some exceptions, class I all-trans head-to-tail isoprenyl pyrophosphate synthases such as FPPS and GGPPS are all structurally very similar proteins with the same basic protein folds and most forming homodimers (Tachibana et al. 1993; Liang et al. 2002; Sun et al. 2005; Kavanagh et al. 2006; Chang et al. 2006; Oldfield and Lin 2012) (Fig. 3). They share substrates with the same basic structure, utilizing IPP as prenyl acceptor with an isoprenyl pyrophosphate donor such as DMAPP to form longer isoprenoid polymers of a specific length depending on the enzyme (Wang and Ohnuma 1999).

The first IPPS structure to be solved was that of avian FPPS (Tarshis et al. 1996); since various other IPPS structures have been determined. The GGPPS crystal structures solved to date are from human, *Plasmodium vivax, Saccharomyces cerevisiae, Oryza sativa*, *Arabidopsis thaliana* and the archaeon *Geoglobus acetivorans* (Guo et al. 2007; Artz et al. 2011; Wang et al. 2016; Zhou et al. 2017; Lacbay et al. 2018; Petrova et al. 2018).

Sequence alignments revealed several highly conserved elements such as the first- and second-aspartate-rich motif (FARM and SARM, D-D-x(2)-D or D-D-x(4)-D) (Ashby and Edwards 1990; Chen et al. 1994). The FARM and SARM (along with other conserved residues) were found to be essential for catalysis and coordinate at least two Mg^2+^ ions (Joly and Edwards 1993; Song and Poulter 1994; Liang 2009; Chang et al. 2012). Studies employing phylogenetic analysis with mutagenesis revealed three different types of GGPPS with a distinct phylogenetic distribution and molecular approach toward their product length specificity [see (Wang and Ohnuma 1999; Feng et al. 2020) and references therein].

Archaeal GGPPS (type-I) have bulky aromatic limiter residues on the fifth position N-terminal to the FARM without any inserted residues in the FARM. The region between the FARM and the fifth position N-terminal being coined the chain-length determination region and is located on helix αD, one of the helices composing the product elongation cavity. Mutational experiments on this region revealed that product chain length could be significantly altered (Tachibana et al. 1993, 2000; Ohnuma et al. 1996a, 1997, 1998a, b). GGPPS from photosynthetic organisms such as plants, algae and cyanobacteria (type-II) have been less intensively studied and have smaller residues on the fourth and fifth position before the FARM, such as serine, alanine and methionine. Additionally, these enzymes contain an insertion of two amino acid residues in the FARM, the first of which is a highly conserved proline residue (Wang et al. 2016; Feng et al. 2020). GGPPS found in mammals and fungi (type-III) have recently attracted renewed interest as a pharmaceutical target in humans (Chen et al. 2008; Liang 2009; Lacbay et al. 2018; Lisnyansky et al. 2018). Type-III GGPPS contain similarly small residues at fourth and fifth positions N-terminal to the FARM compared to type-II GGPPS without any inserted residues in the FARM (Hemmi et al. 2003). However, type-III GGPPS were found to employ a different region for chain-length determination and contain a conserved histidine at 2 positions N-terminal to the G(Q/E) motif located on helix αF which, like the FARM motif, is one of the three helices that form the product elongation pocket (Kavanagh et al. 2006; Chang et al. 2006). Group I, group II and long-chain isoprenyl diphosphate synthases contain a variety of smaller residues at this position. Mutagenesis of the conserved histidine to alanine resulted in the longer GFPP product. Moreover, additional alanine mutations of residues located at the 2 and 3 position N-terminal to this location, that approximately corresponds to the pitch of an α-helix, resulted in the formation of even longer products (Hemmi et al. 2003). Similar findings were observed with mutational studies employed on other closely related enzymes (Tarshis et al. 1996; Ohnuma et al. 1998b). In essence, the positioning of larger residues along the product elongation cavity limits determines the final product chain length, reminiscent to the function of a hydrocarbon ruler (Ahn et al. 2004).

Taken the mutagenesis data on the GGPPS types together, a study on type-II GGPPS proposed a unifying “three-floor” model which explains the molecular basis on final product chain-length determination of short-chain IPPS (Wang et al. 2016). The positioning of large- or medium-sized residues at positions corresponding to particular “floors” in any of the helices that make up the product elongation cavity (αD, αE or αF, Fig. 3b) limits the final product size. Large residues on the “first-floor” limit the enzyme to FPP as the final product, with medium residues on the “first-floor” or large residues on the “second-floor” limiting to GGPP and large residues on the “third-floor” resulting in a GFPP end-product (Wang et al. 2016; Feng et al. 2020).

#### Formation of the first ether bond to glycerol-1-phosphate

Prenyltransferases are responsible for the formation of the ether bonds in archaeal phospholipid biosynthesis. The first ether bond formation is catalyzed by geranylgeranylglycerol phosphate synthase (GGGPS) and involves the formation of an ether bond between glycerol-1-phosphate (G1P) and GGPP which results in geranylgeranylglycerol phosphate (GGGP) (Fig. 1). This reaction marks the first committing step into the biosynthesis of archaeal phospholipids. Presumably, this biosynthesis step occurs in the cytosol as GGGPS enzymes do not contain transmembrane helices and are purified as soluble proteins from the soluble fraction of cell lysates (Payandeh et al. 2006; Peterhoff et al. 2012, 2014; Linde et al. 2018; Nemoto et al. 2019; Blank et al. 2020; Kropp et al. 2021).

In contrast, in bacteria, fatty acids are ester linked to glycerol-3-phosphate (G3P) (Fig. 1). The first enzymes discovered performing this reaction were the enzyme of the PlsB/PlsC pathway. In this pathway, the radyl moiety is attached through transesterification of acyl-ACP (or in some cases also acyl-CoA) to the C-1 position of G3P by the membrane-bound PlsB; resulting in the formation of lyso-phosphatidic acid (LPA) (Cronan and Bell 1974; Bell 1975; Lightner et al. 1980; Yao and Rock 2013). However, the increased availability of genome sequencing techniques revealed that the presence of PlsB is mostly limited to γ-proteobacteria (Parsons and Rock 2013). Instead, most Bacteria use the PlsX/PlsY/PlsC pathway, in which membrane-associated PlsX converts acyl-ACP to an acyl-phosphate intermediate, which subsequently is attached to C-1 of G3P by the integral membrane protein PlsY to form LPA (Larson et al. 1984; Lu et al. 2006; Paoletti et al. 2007; Kim et al. 2009). Despite the similarities in overall organization of the bacterial and archaeal phospholipid biosynthetic pathways, the enzymatic reactions involved are very different. Hence, PlsB, PlsX and PlsY are not members of the prenyltransferase family and thus are also not structurally related to GGGPS.

**Fig. 4:**
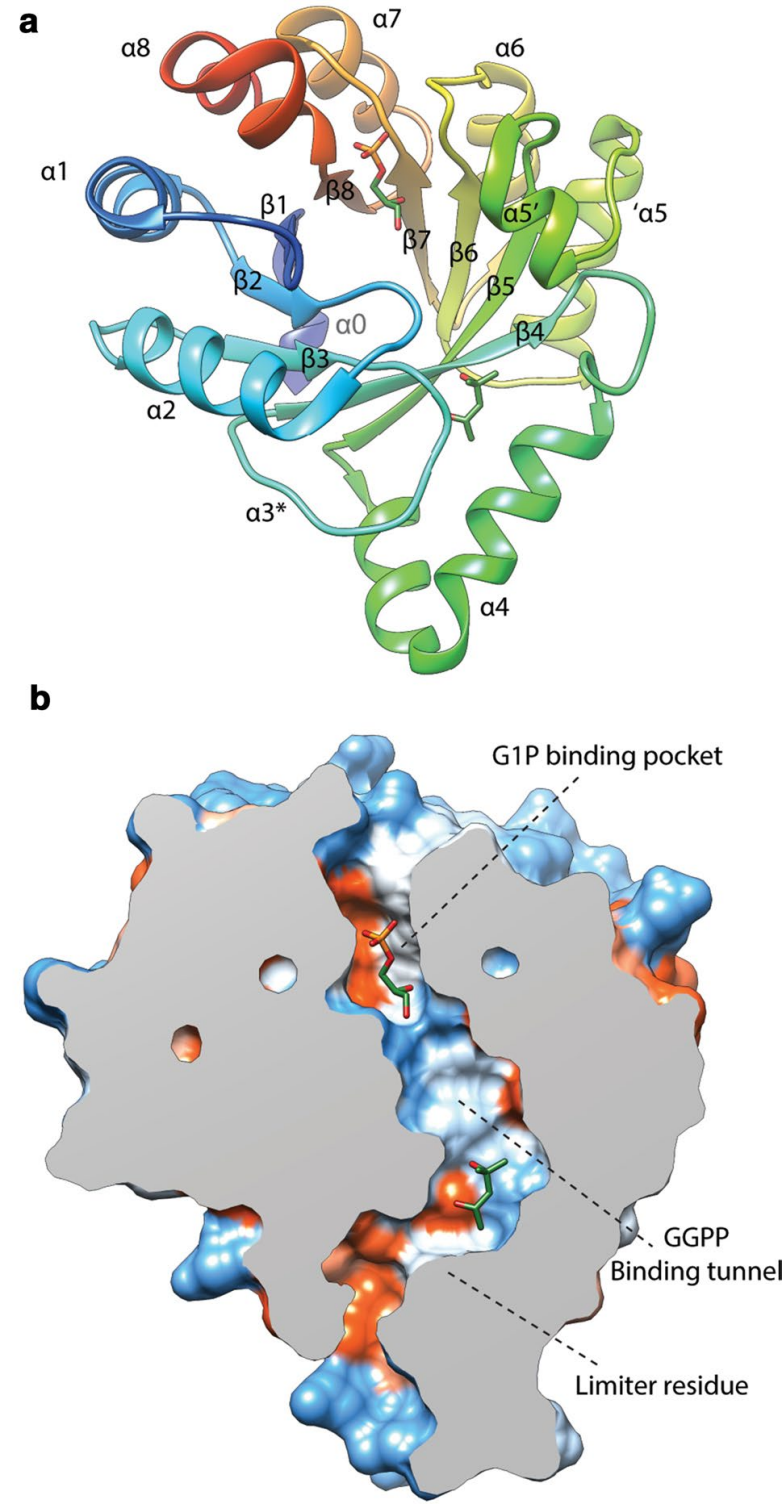
Ribbon view (**a**) and clipped solid-surface view (**b**) of the AfGGGPS crystal structure with bound G1P and 2-methyl-2,4-pentanediol (PDB: 2F6X, (Payandeh et al. 2006)). The surface view is colored according to the Kyte–Doolittle hydrophobicity scale. Red surfaces are hydrophobic, white is of mixed character and blue is hydrophilic

Phylogenetic analysis of members of the GGGPS prenyltransferase sub-family revealed the presence of two distinct groups, group I and group II, which are further subdivided based on archaeal (Ia and IIa) or bacterial origins (Ib and IIb) (Peterhoff et al. 2014). To date, the protein data bank (PDB) contains 6 reported GGGPS crystal structures. Notably, most structures are from thermophilic archaea such as: *Archaeoglobus fulgidu*s (AfGGGPS, group Ia, (Payandeh et al. 2006)), *Methanothermobacter thermoautotrophicus* (MtGGGPS, group IIa, (Peterhoff et al. 2014)), *Thermoplasma acidophilum* (TaGGGPS, group IIa, (Nemoto et al. 2019)) and *Thermoplasma volcanium* (TvGGGPS, group IIa, (Blank et al. 2020)). The structure of a bacterial GGGPS (group IIb, (Peterhoff et al. 2014)) from *Flavobacterium johnsoniae* was also reported (FjGGGPS). The GGGPS crystal structures all show a modified triose phosphate isomerase (TIM-) barrel structure in which an eight-stranded parallel β-barrel with a tightly packed hydrophobic core is surrounded by α-helices constituting a (βα)_8_-barrel structure (Fig. 4a). Characteristic GGGPS TIM-barrel modifications include an additional helix α0 at the N-terminus. Helix α3 is often replaced by a string or strand without any secondary structure whereas in other cases is modeled as a short helix. Helix α5 is split in which helix α5’ is located on top of the barrel over the active site. Helix α3* has been suggested to act as a “swinging door” or part of a ratchet mechanism to aid in expelling the hydrophobic GGGP product from the hydrophobic GGPP-binding site (Payandeh et al. 2006).

Archaea synthesize phospholipids with the G1P stereochemistry. Experiments with both purified enzyme and crude extracts showed high substrate specificity of GGGPS for G1P over G3P (Zhang and Poulter 1993; Chen et al. 1993; Nemoto et al. 2003a). At high concentration, the *Methanococcus maripaludis* GGGPS can utilize both G1P and G3P, but exhibits a eightfold higher affinity for G1P as compared to G3P (Caforio et al. 2018). The substrate specificity of GGGPS is a key point for the synthesis of lipid cores with the correct stereochemistry and therefore the lipid-divide. The positioning of the G1P phosphate group is facilitated by the TIM-barrel “standard phosphate-binding motif” (Nagano et al. 2002; Vega et al. 2003). This motif interacts with G1P through backbone amino groups and side-chains of loops βα6 (S169, G170), βα7 (G194, G195) and βα8 (V214, G215 and N216) (numbering according to AfGGGPS, group I) (Payandeh et al. 2006). Notably, N216 is replaced by a conserved threonine in group II enzymes. It has been proposed that three strictly conserved tyrosine and glutamate residues are the main factors responsible for both substrate stereospecificity and catalytic activity in both group I and group II enzymes (Y124/Y165/E167 and Y134/Y178/E180 in AfGGGPS and MtGGGPS, respectively) (Payandeh et al. 2006; Ren et al. 2013; Peterhoff et al. 2014). Of these three residues, the glutamate could facilitate prenyl acceptance by polarizing the G1P C-3 hydroxyl group (Peterhoff et al. 2014). The current proposed reaction mechanism for the GGGPS family involves the ionization of GGPP to form an allylic geranylgeranyl carbocation through removal of its pyrophosphate moiety by the Mg^2+^ ion and subsequent nucleophilic attack of the polarized C-3 hydroxyl group of G1P on C-1 of the geranylgeranyl carbocation forming the ether bond (Payandeh and Pai 2007; Ren et al. 2013; Peterhoff et al. 2014; Blank et al. 2020). For enzymatic activity, the enzyme needs to bind the hydrophobic polyprenyl substrate of a specific length and the diphosphate group should be oriented in such a way that it can be coupled to the C-3 hydroxyl oxygen of G1P. The diphosphate group of GGPP has been suggested to bind in complex with the Mg^2+^ ion which is coordinated by at least an essential conserved D13 and possibly by T37 in AfGGGPS (D25 and possibly E27/E28 and S54 in MtGGGPS) as shown by mutagenesis experiments and as seen in other prenyl transferases (Payandeh et al. 2006; Payandeh and Pai 2007; Ren et al. 2012, 2013; Peterhoff et al. 2014). The GGPP-binding pocket is varies considerably between different GGGPS crystal structures. It takes the form of a cleft (Nemoto et al. 2019; Blank et al. 2020), tunnel (Payandeh et al. 2006) or pocket (Peterhoff et al. 2014), with a predominantly hydrophobic or mixed character. In AfGGGPS, the GGPP-binding tunnel has an opening at the polar G1P-binding site and runs over the top of the barrel toward the N-terminus of helix α4a and curves down over β3 and β4 between helix α4a and the loop between β3 and β4 (Helix α3*) (Payandeh et al. 2006; Ren et al. 2013) (Fig. 4b). This tunnel is limited by a bulky limiter residue in helix α4a in group I enzymes (W99 in AfGGGPS) and bears some similarities to “hydrocarbon rulers” found in other acyl- and prenyl transferases such as GGPPS (Wyckoff et al. 1998; Liang et al. 2002; Ahn et al. 2004; Payandeh et al. 2006). In group II enzymes, the limiter residue is positioned on helix α3’ instead of helix α4a and is not as clearly defined as in group I, since smaller residues seem to be involved (proposed to be I90 in FjGGGPS and V86 in MtGGGPS) (Peterhoff et al. 2014).

The prenyltransferase family also contains enzyme subfamilies with different substrate specificity such as the bacterial heptaprenyltransferases (PcrB, “GGGPS” group Ib) which are homologous to archaeal GGGPS. Members of the PcrB sub-family catalyze the transfer of heptaprenyl diphosphate moieties (C_35_) to G1P forming heptaprenylglycerol phosphate in contrast to the shorter tetraprenyl moieties (C_20_) transferred by GGGPS (Guldan et al. 2011; Peterhoff et al. 2012). Being homologs, the structures of these enzymes are quite similar. In addition, the shape and amino acid identity of the G1P-binding pocket was found to be strongly conserved between *A. fulgidus* GGGPS and *Bacillus subtilis* PcrB (Payandeh et al. 2006; Guldan et al. 2011). The difference in substrate specificity between these two subfamilies was found to be caused by the bulky limiter residue at the bottom of the GGPP-binding site in helix α4a of AfGGGPS (W99). For AfGGGPS, the isoprenyl chain length is limited to C_20_ (GGPP), while in the related BsPcrB, this residue is replaced with alanine (A100) and substrate limiting is facilitated by Y104, situated lower on helix α4a to allow for polyprenyl substrates up to C_35_ in length. Interestingly, haloarchaea producing C_20_–C_25_ ether lipids were found to possess two paralogous GGGPS group I enzymes referred to as subgroups IaH1 and IaH2. Haloarchaeal GGGPS IaH1 enzymes contain the prototypical tryptophan limiter residue, whereas IaH2 enzymes have a leucine which might allow for the binding of longer substrates (Peterhoff et al. 2014). These structural elements may form the molecular basis of the presence of C_20_–C_25_ ether lipids in these organisms and supports a previous hypothesis which stipulates that a few mutations could be enough to alter prenyl donor selectivity of GGGPS (Boucher et al. 2004). The crystal structure of the more distantly related MoeO5 prenyltransferase has been solved as well (Ren et al. 2012). MoeO5 shares many structural features with GGGPS and PcrB, such as a similar substrate-binding site. However, the reaction catalyzed by MoeO5 is rather unusual as the prenyl group of FPP is transferred to 3-phosphoglycerate, during which the intramolecular trans-allylic bond of FPP gets converted into a cis-allylic bond.

**Fig. 5:**
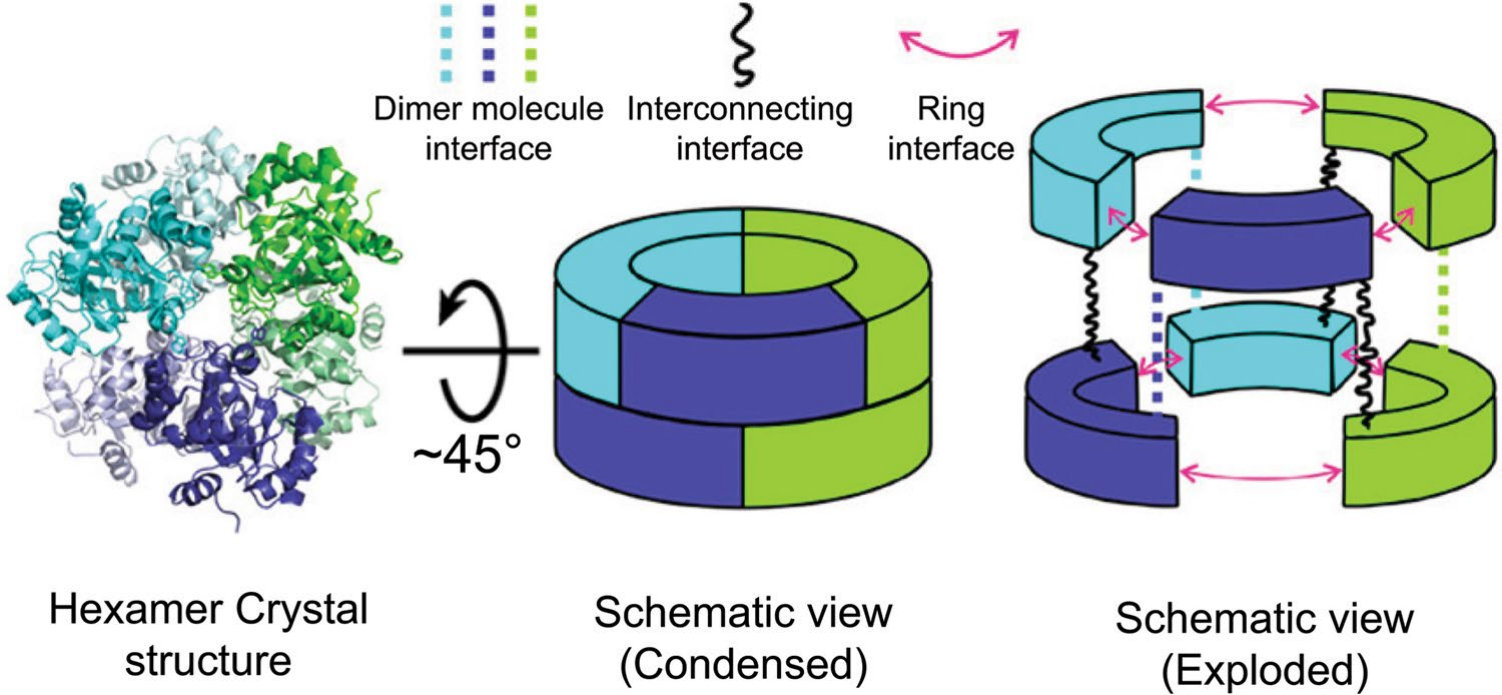
The hexameric configuration of MtGGGPS with a schematic representation of the different interfaces facilitating hexamerization. The dimers are colored green, cyan and purple. Adapted with permission from Linde, et al. (Linde et al. 2018). Copyright 2018 American Chemical Society

GGGPS homologs adopt different oligomerization states, for example: AfGGGPS, TaGGGPS, TvGGGPS and FjGGGPS form dimers (Nemoto et al. 2003a; Payandeh et al. 2006; Peterhoff et al. 2014; Blank et al. 2020), whereas MtGGGPS, *Thermococcus kodakarensis* GGGPS and *Chitinophaga pinensis* GGGPS were found to form hexamers (Peterhoff et al. 2014). A correlation was found between similarity network clustering and the oligomerization state as revealed by selective light scattering and size exclusion chromatography. This showed that GGGPS enzymes belonging to group I form dimers, whereas almost all group II members are expected to form hexamers with the exception of bacterial GGGPS and GGGPS from Thermoplasmatales (Peterhoff et al. 2014; Linde et al. 2018; Kropp et al. 2021). Most group II enzymes were found to have a conserved aromatic anchor residue (W, Y, F) on helix α5 participating in a cation–pi interaction which was shown to be essential for hexamerization (Peterhoff et al. 2014; Linde et al. 2018). Recently, it was shown (Kropp et al. 2021) that most other group II GGGPS, which did not have the typically conserved W, Y or F aromatic anchor and thus were initially expected to form dimers, contained a histidine residue instead. This histidine was shown to be able to participate in cation–pi interactions, and therefore, those group II proteins are also expected to form hexamers (Kropp et al. 2021). The hexameric state of GGGPS has previously been described as a trimer of dimers and can be visualized as tilted dimers interlocking at a 120-degree angle forming a stacked “upper and lower” ring of three polypeptides of different dimers each (Fig. 5). Thus, three different interfaces connect the subunits: the symmetrical dimer interface, which is found in natively dimeric GGGPS and is diagonal to the horizontal plane of the two-stacked-ring hexameric complex connecting the rings together; the ring interface, which contains the aromatic anchor residue and laterally connects the three polypeptides of three different dimers forming a ring; and the interconnection interface, which is also situated between three polypeptides of the three different dimers vertically connecting the two rings forming the stacked ring shape. Studies looking at residues which play a role in the formation of the hexameric complex found than an aromatic anchor residue in helix α5’ to be essential to the formation of hexameric GGGPS complexes (Peterhoff et al. 2014; Linde et al. 2018; Kropp et al. 2021).

Oligomerization has been regarded as a factor enhancing thermostability (Sterner and Liebl 2001; Vieille and Zeikus 2001). Linde et al. investigated the effect of oligomerization on the thermostability of MtGGGPS and found that the temperature of the first denaturation transition step, which correlates with a loss of catalytic activity, increased with a higher order oligomeric state (Linde et al. 2018). This indicates that the hexameric state confers increased thermostability. Furthermore, a dimeric MtGGGPS mutant had its catalytic efficiency drastically reduced (K_m_ (G1P) value × 50 higher), indicating that hexamerization stabilizes the G1P-binding pocket (Kropp et al. 2021). MD simulations at denaturing temperatures revealed that the flexibility of four regions was significantly higher than in other parts of the protein compared to non-denaturing temperature simulations. Three of these regions, Helix α4, loop βα6 and helix α5’, are involved in substrate binding, explaining the loss of activity during the first denaturation transition. However, the region situated in helix α5’ is of particular interest as this region normally covers the GGPP-binding site but also contains the aromatic anchor residue which is part of the ring interface and would stabilize this region if the complex would be in the hexameric quaternary state. Thus, it has been suggested that hexamerization allows for a balance between more flexibility (possibly related to activity at lower temperatures) and thermostability. In contrast, not all hyperthermophilic GGGPS adopt hexameric oligomerization states, and thus, it seems not a strictly essential feature for hyperthermophilic GGGPS enzymes. Furthermore, a recent ancestral sequence reconstruction (ASR) analysis study (Blank et al. 2020) which focused on the aromatic anchor residues (specifically W, Y and F only) showed that these residues are the result of convergent evolution without temperature being the primary driving factor for this development. This is in contrast another study which argues that hexamerization evolved as a general thermostability feature in group II GGGPS (Kropp et al. 2021).

#### Formation of the second ether bond to form the diether

**Fig. 6:**
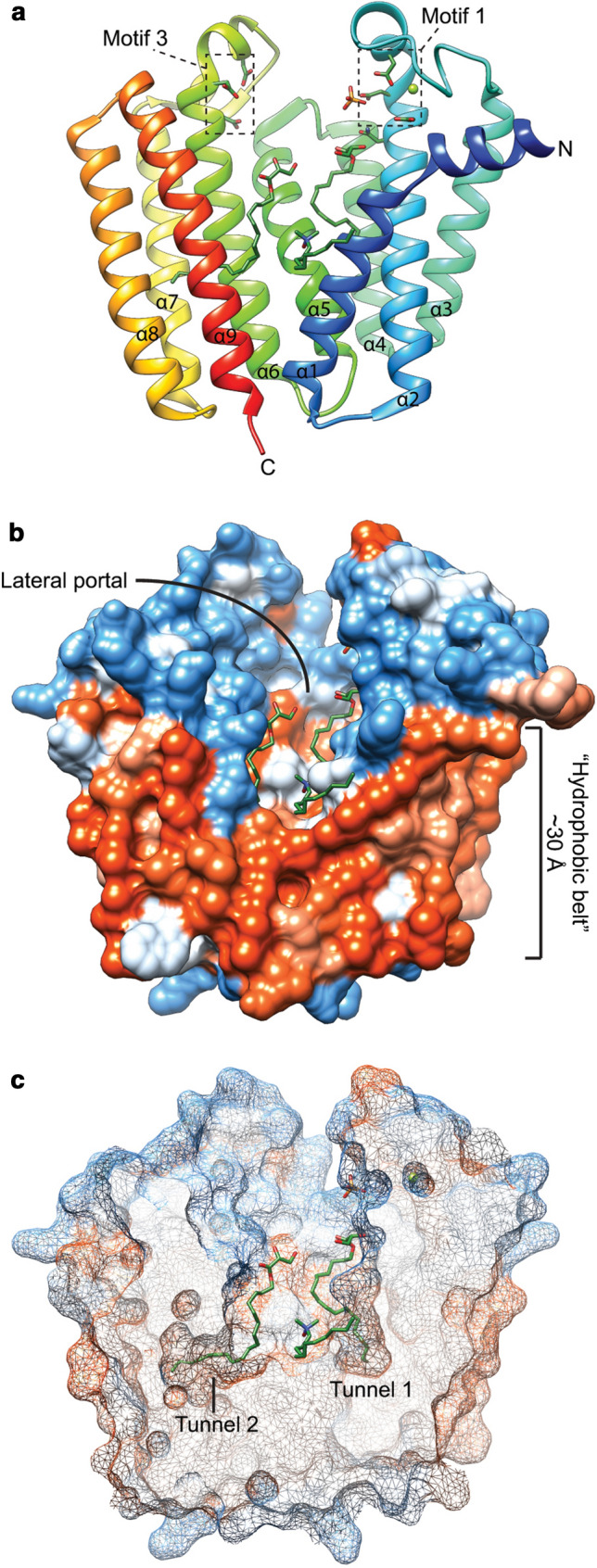
Ribbon view (**a**), solid-surface view (**b**) and clipped mesh-surface view of the MjDGGGPS crystal structure with bound [octadec-9-enyl]-2,3-hydroxy-propanoate and lauryldimethylamine oxide (LDAO) (PDB: 6M31, (Ren et al. 2020)). The surface views are colored according to the Kyte–Doolittle hydrophobicity scale. Red surfaces are hydrophobic, white is mixed character and blue is hydrophilic. Mg2 + ions are shown as green spheres. In panel A the aspartic acid residues of motif 1 and 3, and asparagine 62 are shown in liquorice style

The second ether bond formation between GGGP and GGPP results in di-geranylgeranylglycerol phosphate (DGGGP) and is catalyzed by di-geranylgeranylglycerol phosphate synthase (DGGGPS) (Fig. 1), a member of the UbiA superfamily. Structural analysis shows that MjDGGGPS is an integral membrane protein with an N-terminal amphipathic helix, followed by 9 transmembrane helices forming a central cavity containing two hydrophobic lipid-binding tunnels (tunnel 1 and tunnel 2) and a cytoplasmic opening with a lateral portal into the membrane (Ren et al. 2020) (Fig. 6). The cytoplasmic opening crowning the central cavity is referred to as the cytoplasmic domain and contains three loop/helix structures capping the cytoplasmic opening. The bottom of the lateral portal is surrounded by a pronounced “belt” of hydrophobic residues spanning ~ 30 Å in overall thickness covering the entire circumference of the enzyme and likely corresponds to the positioning of the phospholipid bilayer of the host organism *Methanocaldococcus jannaschii* (Fig. 6b).

In Bacteria, the analogous reaction to the formation of the second *ether* bond by DGGGPS is the formation of the second *ester* bond facilitated by the membrane-associated protein PlsC (Coleman 1990, 1992; Robertson et al. 2017). PlsC facilitates the transesterification of acyl-ACP (sometimes acyl-CoA is accepted) with LPA resulting in the formation of PA. Similar to PlsB, PlsC belongs to family of phospholipid acyltransferases. Hence, PlsC is not structurally or mechanistically related to DGGGPS. Hence, the early steps in the phospholipid biosynthetic pathways in Archaea and Bacteria must have evolved independently.

Members of the UbiA superfamily generally transfer a polyprenyl group to hydrophobic acceptor molecules. However, the accepting groups and overall structure of acceptor molecules vary widely, including: An ethylene carbon in protoheme (Saiki et al. 1992, 1993), a carbon as part of an aromatic ring for the synthesis of quinones or tocopheroles (Siebert et al. 1992; Suvarna et al. 1998; Collakova and DellaPenna 2001; Savidge et al. 2002), the carboxyl carbon in a propionate side chain of chlorophyllides (Oster and Rüdiger 1997), or the C-2 of the glycerol backbone containing a hydroxyl group in GGGP (Hemmi et al. 2004; Roy et al. 2010; Ren et al. 2020). Remarkably, phylogenetic analysis by Hemmi et al. (Hemmi et al. 2004) revealed that UbiA enzymes cluster according to their prenyl acceptor substrate structure, and not prenyl donor substrate structure. This indicates that the conserved motifs are likely related to recognition of the prenyl donor and catalytic mechanism. Indeed, these structural elements in MjDGGGPS resemble that of soluble prenyl transferases such as GGPPS and FPPS (Hosfield et al. 2004; Kavanagh et al. 2006).

The molecular mechanism of the UbiA superfamily was explored by studying the activity of MjDGGGPS mutants (Ren et al. 2020). Members of the DGGGPS family contain two conserved Asp-rich motifs with the D_66_-x(3)-D_70_ (motif 1) and D_183_-x(2)-D_187_-x(3)-D_190_ (motif 3) motifs as well as a conserved Y_125_-x(5)-K_130_ (motif 2) motif (MjDGGGPS numbering). Other members of the UbiA superfamily such as CoQ2, UbiA, MenA, and Cox10 also contain similar motifs. These highly conserved motifs are each located on one of the capping loop/helix structures, indicating that these structures are directly or indirectly involved in catalysis. This idea is further reinforced by the Mg^2+^ ion that is coordinated by motif 1 and the positioning of the pyrophosphate moiety of DMAPP, free phosphates and 2,3-hydroxy-propanoate “lipid backbone” of the co-crystallized [octadec-9-enyl]-2,3-hydroxy-propanoate. MjDGGGPS activity assays revealed that alanine mutants of D66, D70, D180, D183 and D187 strongly reduced MjDGGGPS activity, indicating that these residues indeed perform critical roles in catalysis. Moreover, in line with other prenyltransferases, MjDGGGPS prefers Mg^2+^ over other divalent cations, while EDTA abolishes the activity, showing that divalent cations are essential for enzymatic activity. It was proposed that D66 and D70 are responsible for coordination of an Mg^2+^ ion whereas D180, D183 and D187 can either coordinate another Mg^2+^ ion or the pyrophosphate moiety by hydrogen bonding. In this context, it is noteworthy that the previously reported *A. fulgidus* UbiA structures show two Mg^2+^ or Cd^2+^ ions bound in regions corresponding to motif 1 and motif 3 (Huang et al. 2014). The structure of DGGGPS suggests that the orientation of the hydroxyl group on C-2 of GGGP could be important for catalysis as this is where the coupling of GGGP with the C-1 of GGPP happens. This is consistent with the chirality of the GGGP glycerol backbone produced by GGGPS in vivo. However, DGGGPS has been shown to accept G3P-based GGGP, as well (Zhang et al. 2006). Hence, it appears that this enzyme is less critical in defining the overall chirality of the phospholipid backbone compared to GGGPS.

Lipid-binding experiments showed that the motif 1 (N62A, D66A, D70A) and motif 3 (D180A, D183A and D187A) mutations only had negligible effect on the binding of GGPP, whereas GGGP binding was impaired to varying degrees, indicating that these motifs are important for GGGP binding and catalysis. Mutations of residues lining “lipid-binding tunnel 2”, I29, S171 and F148, abolished GGPP, but not GGGP binding. These data defined the substrate-binding positions in the enzyme (GGPP in tunnel 2 and GGGP in tunnel 1) and indicates Mg^2+^ might not only have a catalytic role but might play a role in GGGP binding or positioning as well. This was further corroborated by MD simulations, suggesting that GGGP binds less deep in the central cavity of the enzyme as compared to GGPP.

The N62A and Y125A mutants showed significantly decreased activity, without a large decrease in substrate affinity, indicating that these residues do not play a major role in catalysis or substrate binding, but might aid in properly orienting the substrates for catalysis. N62 and Y125 are expected to be located in close proximity of the glycerol backbone of GGGP hinting at a role in correct positioning of the prenyl acceptor (glycerol backbone C-2) to be accessible for the prenyl donor (GGPP, C-1). N62 is also located near the Mg^2+^ ion bound by motif 1, possibly aiding in Mg^2+^ positioning.

Interestingly, residue N62 in MjDGGGPS corresponds to N102 in the human UBIAD1 and is located along the central cavity and cytoplasmic opening (Fig. 6a). Mutations in that residue and other nearby residues have been implicated in the occurrence of Schnyder corneal dystrophy (SCD) and other diseases caused by quinone deficiency in humans (Nickerson et al. 2010; 2013). This association was further emphasized by the lack of activity of the corresponding mutants of homologously expressed CoQ2 (from *S. cerevisiae*) and heterologously expressed Human UBIAD1 (Ren et al. 2020). The study of MjDGGGPS was the first study of an UbiA superfamily member that could directly couple enzymatic activity to a crystal structure.

The exact catalytic mechanism of DGGGPS remains to be confirmed. However, because of the similarity in conserved motifs, tertiary structure and prenyl donor substrates, DGGGPS is expected to employ a similar catalytic mechanism as that of soluble prenyltransferases and other members of the UbiA superfamily (Cheng and Li 2014; Huang et al. 2014; Ren et al. 2020).

### Polar head group activation and modification

The next step in archaeal phospholipid biosynthesis is the activation of DGGGP with CTP for polar headgroup attachment, yielding CMP-DGGGP (CDP- archaeol^1^)) (Fig. 1). CDP-archaeol is a key intermediate in the pathway and the precursor for phospholipid headgroup differentiation. The aforementioned reaction is achieved by the cytidyl-diphosphate-archaeol synthase enzyme CarS. The CTP-transferase reaction of CarS in Archaea is analogous to CDP-DAG formation in Bacteria, but is achieved by a different, only distantly related enzyme (CdsA), although both enzymes belong to the CTP-transferase superfamily (Jain et al. 2014). The activity of this enzyme was first demonstrated in crude lysates of *M. thermoautotrophicus* using synthetic substrates (Morii et al. 2000). Activity assays using the crude membrane fraction of *M. thermoautotrophicus* showed a strong specificity toward the conversion of lipid substrates containing unsaturated geranylgeranyl groups, such as DGGGP over saturated archaetidic acids (AA) or fatty acid-based lipids. However, no strong selectivity was reported for DGGGP analogs containing ester instead of ether bonds or containing G3P—instead of G1P-backbone stereochemistry. Furthermore, catalytic studies with purified *Archaeoglobus fulgidus* CarS (AfCarS) confirm the high selectivity for DGGGP compared to bacterial phosphatidic acids (PA), while the *Escherichia coli* CdsA shows significantly more activity on PA compared to DGGGP (Jain et al. 2014). In addition, the archaeal AfCarS could not complement the conditional growth defect of an *E. coli cdsA* mutant strain. Taken together, these studies show that CarS and CdsA effectively differentiate between archaeal and bacterial substrates and uphold the lipid-divide. However, the molecular basis of CarS substrate specificity between PA and DGGGP has not been studied in vitro in detail with purified enzyme instead of a crude membrane fraction (Morii et al. 2000).

**Fig. 7:**
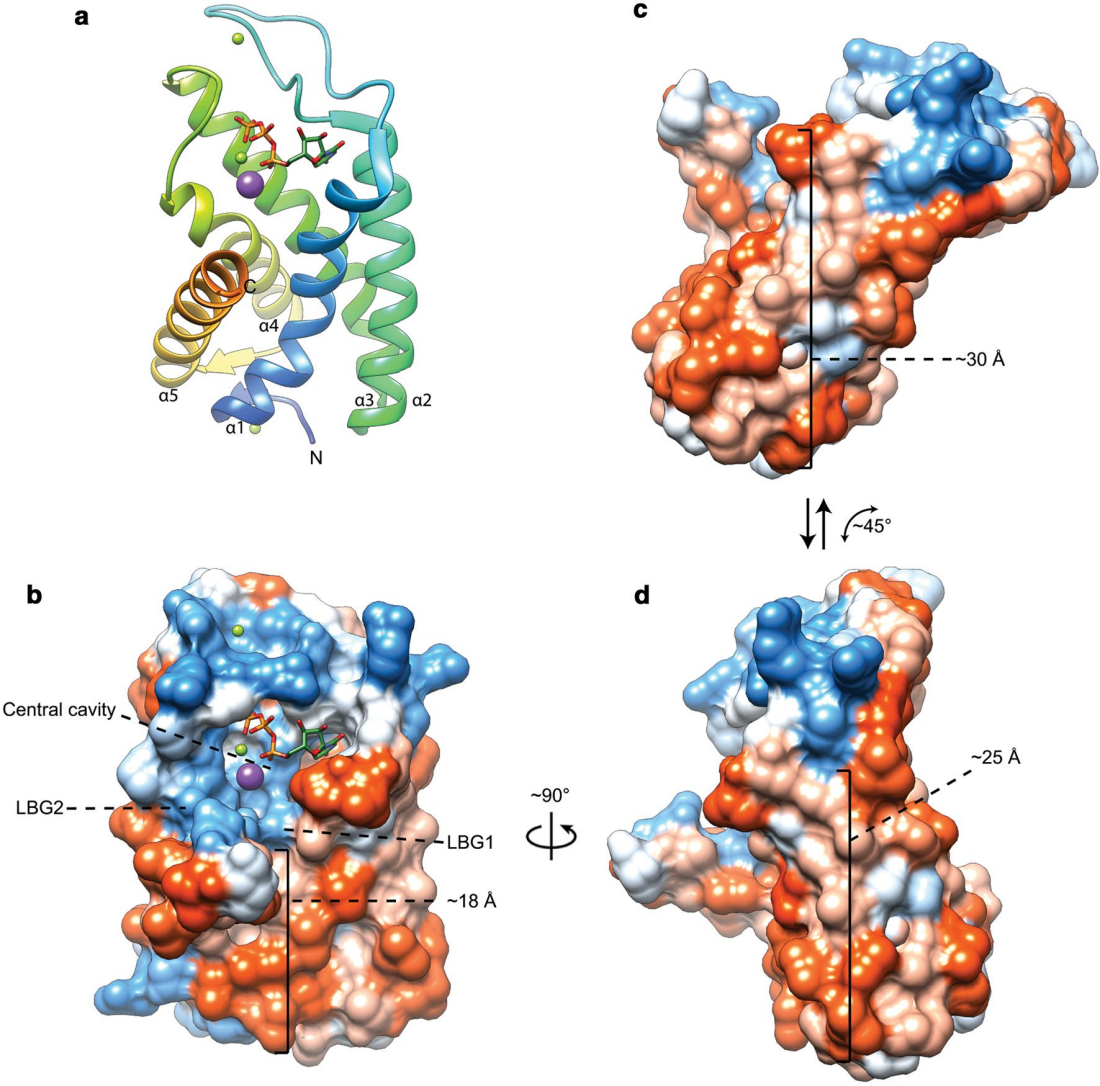
Ribbon view (**a**) and solid-surface view (**b**, **c**, **d**) of the ApCarS crystal structure with bound CTP (PDB: 5GUF, (Ren et al. 2017)). The surface views are colored according to the Kyte–Doolittle hydrophobicity scale. Panels c and d illustrate the suggested tilting motion where ApCarS could adopt a position that would favor CTP-binding/PPi release (**c**) and a position that would encourage lipid–substrate binding or product release (**d**). Red surfaces are hydrophobic, white is of mixed character and blue is hydrophilic. Mg2 + and K + ions are shown as green and purple spheres, respectively

To date, only the crystal structure of *Aeropyrum pernix* CarS (ApCarS) has been reported (Ren et al. 2017). Five transmembrane helices provide the primary structural elements with two cytoplasmic loops forming a polar cytoplasmic domain (Fig. 7a). The cytoplasmic domain contains a polar central cavity which in turn harbors a small hydrophobic cytidyl-binding pocket on one side and a small polar phosphate-binding pocket on the other side (Fig. 7b). The central cavity is positioned in a cup shaped by the termini of transmembrane helices α1 to α4 where transmembrane helix α5 only seems to loosely cover the central cavity from the side. The rest of the central cavity is comprised by the two cytoplasmic loops forming the top half of the cavity. Despite being an integral membrane protein, ApCarS does not have hydrophobic tunnels to accommodate isoprenoid tails like DGGGPS but rather has two lipid-binding grooves (LBG) split by helix α5. Moreover, the polar cavity is located around the top of the hydrophobic “belt” around the enzyme resulting in a somewhat thinner belt section directly adjacent to the active site (~ 18 Å, Fig. 7b). The rear outer surface of the polar cavity is remarkably hydrophobic as well resulting in a ticker hydrophobic belt (~ 30 Å, Fig. 7c). This might be an indication that the enzyme is able to tilt backward into the membrane, possibly as a mechanism to bind CTP or discharge PPi (Fig. 7c and 7d). Or, depending on perspective, the enzyme may tilt forward to bind DGGGP which would be present in the membrane. Alternatively, this large hydrophobic surface on the rear of the enzyme could serve as a surface for interaction with other proteins. The reaction mechanism of CarS is proposed to be comprised of 3 steps: polarization of the CTP α-phosphate, transesterification by nucleophilic attack of the negatively charged phosphate oxygen of DGGGP on the CTP α-phosphate followed by product release (Ren et al. 2017).

ApCarS was co-crystallized with CTP that is bound in the polar cavity with the pyrimidine moiety pointing into the hydrophobic pocket and with the triphosphate moiety being coordinated by a Mg^2+^ and K^+^ ion (Ren et al. 2017). This agrees with the observation that CarS is Mg^2+^ dependent, with its activity being abolished in the presence of EDTA. Furthermore, the presence of K^+^ ions considerably increases the activity of ApCarS (Jain et al. 2014; Ren et al. 2017). The positioning of the triphosphate moiety is of particular interest as the γ- and β-phosphate are buried in a highly polarized pocket, whereas the α-phosphate is mostly solvent exposed and accessible for a nucleophilic attack by the DGGGP acceptor substrate.

DGGGP binds to CarS through hydrophobic interactions with two grooves lined with conserved hydrophobic residues and through hydrogen bonding with polar residues in the central cavity. These two lipid-binding grooves are primarily formed by helix α5 loosely covering the central cavity containing the CTP and Mg^2+^-binding sites (Fig. 7b). Interestingly, the *Thermotoga maritima* CdsA structure (TmCdsA, PDB: 4Q2E) around the active site aligns well with the structure of CarS. The largest structural difference seems to be caused by a different positioning of ApCarS helix α5 compared to the corresponding helix in TmCdsA (Helix α1), resulting in a different shape of the hydrophobic groove. The lipid-binding grooves in ApCarS are overall wider compared to the groove in TmCdsA, rendering TmCdsA unable to properly accommodate the bulkier isoprenoid chains of archaeal phospholipids compared to the less-bulky bacterial fatty acyl-based phospholipids (Ren et al. 2017). This is likely the reason why *E. coli* CdsA does not readily accept DGGGP as a substrate (Morii et al. 2000).

CdsA and CarS are both members of the CTP-transferase family but only share a low degree of homology, highlighting that the enzymes are evolutionary distant and have diverged significantly from one another forming two separate CTP-transferase subfamilies. Even though the overall structures of CarS and CdsA have diverged significantly, the enzyme catalytic core and structural elements involved in the binding of substrates have been conserved reasonably well.

### Phospholipid polar headgroup modification

**Fig. 8:**
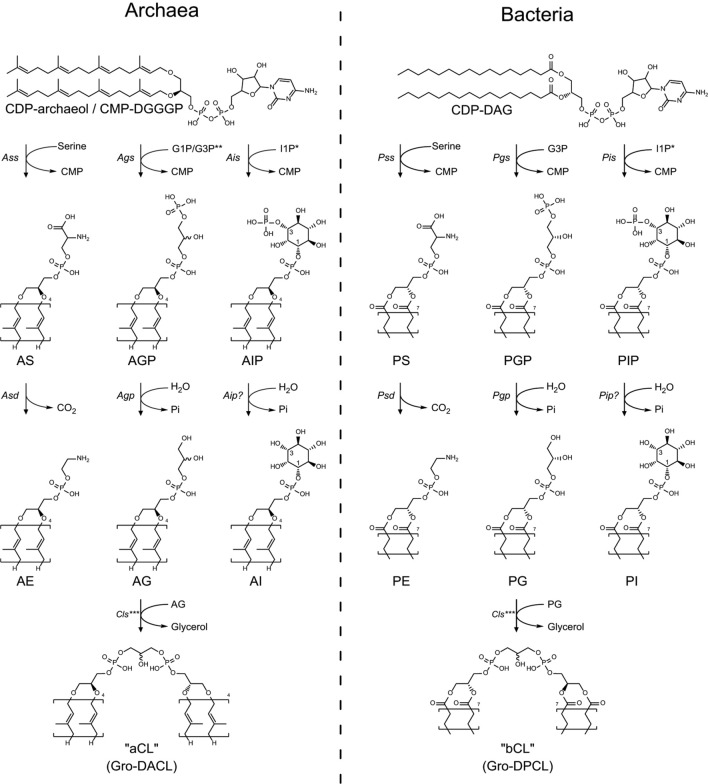
Phospholipid headgroup differentiation pathways of selected common phospholipid headgroups in Archaea and/or Bacteria. * 1L-myo-inositol-1-phosphate. The numbering changes once bound to the phosphatidyl phosphate. ** No information is available on whether archaeal Pgs (Ags) shows any specificity toward G1P or G3P. *** For clarity, only the prototypical addition of two AG/PG molecules to form a single archaeal- or bacterial-CL molecule (aCL/bCL) and glycerol is shown. It is not known whether the substrates for aCL formation are required to be fully saturated or whether saturation can still take place after aCL formation

The now-widespread use of liquid chromatography coupled to high-resolution MS (LC–MS) techniques has uncovered an unprecedented diversity of lipids in the domain of Archaea (Hoefs et al. 1997; Morii et al. 1998; Schouten et al. 2000; Boumann et al. 2006; Wörmer et al. 2013; Jensen et al. 2015a; Knappy et al. 2015; Becker et al. 2016; Bale et al. 2019; Law and Zhang 2019). As such, there are many possible phospholipid headgroup modifications after the phosphate moiety of DGGGP has been activated by CarS. Thus, headgroup modification is discussed from the perspective of well-studied bacteria such as *E. coli* or *B. subtilis* of which homologous enzymes have been identified in Archaea (Daiyasu et al. 2005; Lombard et al. 2012b) (Fig. 8).

The first enzymes involved in phospholipid headgroup modification after CDP-archaeol formation generally belong to the CDP-alcohol phosphatidyl transferase (CAPT) family. At this point, the phospholipid biosynthesis pathway diversifies and the enzymes responsible for the synthesis of particular phospholipids becomes species specific. Members of this family catalyze the displacement of CMP from the CDP-alcohol with another alcohol, such as: glycerol-3-phosphate, *myo*-inositol phosphate or serine. This enzymatic family is rather diverse in phylogenetic distribution and sequence length. However, they all share a hallmark CAPT-motif (D-x(2)-D-G-x(2)-A-R-x(7,8)-G-x(3)-D-x(3)-D, PROSITE PS00379) responsible for coordination of one or two catalytically important divalent metal ions (Williams and McMaster 1998; Hulo et al. 2004; Daiyasu et al. 2005; Sciara et al. 2014; Nogly et al. 2014; Caforio et al. 2015; Grāve et al. 2019). Most members of the CAPT family employ an ordered sequential Bi–Bi mechanism for which both substrates have to bind before catalysis can take place and a product can be released (Cleland 1963; Hirabayashi et al. 1976; Bae-Lee and Carman 1984; Dutt and Dowhan 1985; Aktas et al. 2014). Phylogenetic analysis revealed that bacterial and archaeal CAPTs cluster into groups according to their substrate specificity, for example: G3P, *myo*-inositol phosphate or serine (Morii and Koga 2003; Daiyasu et al. 2005; Morii et al. 2009; Grāve et al. 2019). Representatives are found for all three groups in Bacteria, but only in the latter, two groups are found in archaea. Due to the similarity in sequences between archaeal *myo*-inositol phosphate transferases and G3P transferases, the archaeal G3P transferases cluster into the *myo*-inositol-phosphate transferase group (Daiyasu et al. 2005).

#### Phosphatidylethanolamine synthesis

Phosphatidylethanolamine (PE) forms a major portion of the phospholipids in the membranes of both *E. coli*, *B. subtilis* and many other bacteria (Gidden et al. 2009). In bacteria, PE is usually synthesized from CDP-DAG in a two-step pathway (Fig. 8): The CMP moiety in CDP-DAG is exchanged for L-serine by phosphatidylserine synthase (Pss), typically a CAPT^2^, to form phosphatidylserine (PS). PS is subsequently decarboxylated by phosphatidylserine decarboxylase (Psd) resulting in PE (Dutt and Dowhan 1981; Okada et al. 1994; Nishibori et al. 2005). Homologs of these enzymes, archaetidylserine synthase (Ass) and archaetidylethanolamine decarboxylase (Asd), have been identified in some archaeal species, indicating that the biosynthetic pathway in these organisms is analogous to that of bacteria (Morii and Koga 2003; Daiyasu et al. 2005; Lombard et al. 2012b; Abdul-Halim et al. 2020). However, to date, no archaeal Ass or Asd structures have been published and most insights in this family of proteins have been obtained with the bacterial enzymes.

A structure of Pss from *Haemophilus influenzae* (PDB: 3HSI) has been deposited in PDB, but has not further been described. Recently, the structure of *E. coli* Psd has been solved (Watanabe et al. 2020; Cho et al. 2021). Initially, EcPsd is transcribed as a single polypeptide. This pro-peptide undergoes an autocatalytic maturation event entailing proteolytic cleavage of a highly conserved LGST motif of the pro-peptide into a smaller α-subunit and larger membrane-associated β-subunit (Choi et al. 2015; Ogunbona et al. 2017). Psds are unusual decarboxylases as they contain a unconventional pyruvoyl prosthetic group in the active site (Satre and Kennedy 1978). The serine-protease autoproteolytic mechanism leaves a dehydroalanine residue as the N-terminus of the *α*-subunit in lieu of the original serine of the pro-peptide LGST motif (Li and Dowhan 1988). This dehydroalanine residue is rehydrated and consequently eliminates ammonia to form the pyruvoyl prosthetic group required for enzymatic activity (Li and Dowhan 1990; Ekstrom et al. 2001).

**Fig. 9:**
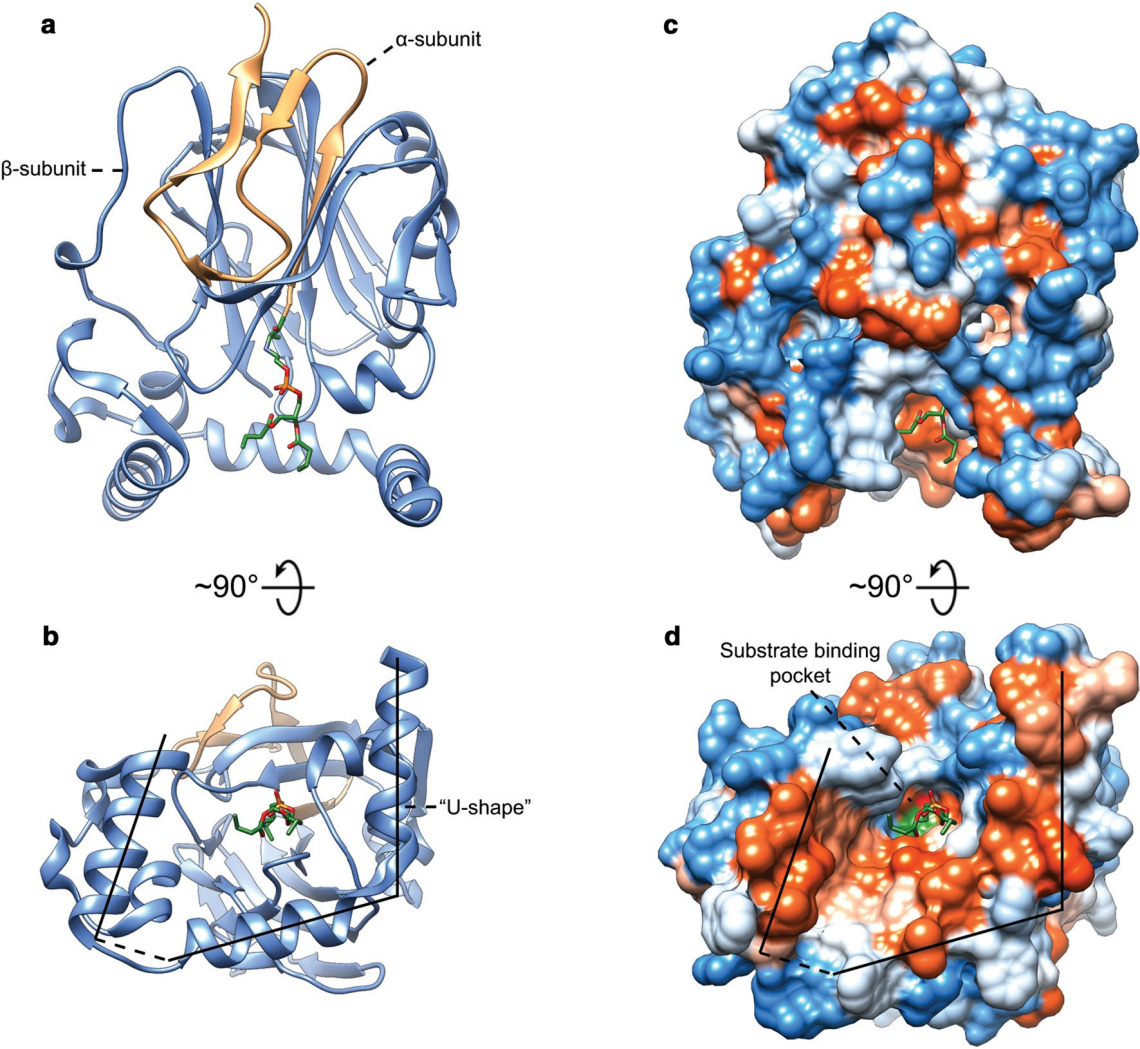
Ribbon view (**a**, **b**) and solid-surface view (**c**, **d**) of a monomer of the EcPsd crystal structure with bound PE (PDB: 6L07, (Watanabe et al. 2020)). The peptide chains corresponding to α- and β-subunits in the ribbon view are colored sandy brown and cornflower blue, respectively. The surface views are colored according to the Kyte–Doolittle hydrophobicity scale where red surfaces are hydrophobic, white is of mixed character and blue is hydrophilic

The EcPsd *α*- and *β*-subunits together create one globular fold consisting of 7 *α*-helices and 18 *β*-strands in total. The structural core of the protein is formed by the main β-sheet-like structure consisting of 7 *β*-strands (Fig. 9a). One broad side of the main sheet is covered by 4 small α-helices and 2 *β*-strands, while the other broad side is covered by 8 *β*-strands of which 4 strands form a small *β*-sheet-like structure parallel to the main β-sheet. One of the short sides of the main *β*-sheet is crowned by the 3 large N-terminal α-helices with an amphipathic character arranged in a “U”-shape which harbors the substrate-binding pocket and active site and likely peripherally associates the enzymes with the membrane and enables phospholipid substrates to diffuse from the membrane into the active site (Watanabe et al. 2020) (Fig. 9b and 9d). The pyruvoyl moiety is located on the N-terminus of the α-subunit which forms a *β*-strand which is securely stabilized as part of the main *β*-sheet-like structure (Fig. 9a). The rest of the *α*-subunit forms 3 small *β*-strands that interact with the main *β*-sheet-like structure.

The crystal structures of EcPsd with bound PE show that the interactions of the lipid tail are mostly facilitated by non-specific hydrophobic interactions (Fig. 9c and 9d). However, the phosphate moiety seems to engage in hydrogen-bonding interactions with Y137 and the amide backbone of V167. In some structures, the conserved S166 side chain also forms a hydrogen bond with PE (Cho et al. 2021). The conserved residue H144 is in close proximity to the ethanolamine headgroup and is known to play an essential role in the decarboxylase activity; while D90 and H147 are not located in the active site and are likely only involved in protein maturation as suggested by mutagenesis experiments in which mutants of D90, H147 and S254 result in failure of protein maturation (and subsequent loss of activity). Regarding mutagenesis of H144, conflicting results have been reported. One study shows that H144 mutants of EcPsd are significantly impaired in pro-peptide maturation and lost almost all activity, while in another study, apparent autoproteolytic maturation was demonstrated of mutants of H144 and H147 (Watanabe et al. 2020; Cho et al. 2021). This could indicate that the conserved autoproteolytic catalytic residues (except for the conserved serine) might not be absolutely essential and other, non-conserved residues can fill-in and participate in autoproteolysis.

Several archaeal Pss homologs have been identified in archaea and two have been studied (Morii and Koga 2003; Abdul-Halim et al. 2020). Notably, the Pss and Psd homologs of *Haloferax volcanii* (Ass and Asd) play a critical role in S-layer glycoprotein lipidation and the proteolytic processing of archaeosortase A (ArtA) substrates.

Traditionally, phospholipid biosynthesis was seen as homogenous process resulting in phospholipids that are randomly distributed over the membrane. The notion of heterogeneity in the phospholipid membrane, “a mosaic of phospholipids”, was developed some decades ago, and has been further explored in various studies on phospholipid localization and lipid microdomains (Singer and Nicolson 1972; Holthuis et al. 2003; Nishibori et al. 2005; Lingwood and Simons 2010; Sonnino and Prinetti 2012; Jiang et al. 2019). To this day, the heterogeneity of the archaeal phospholipid membrane and the enzymatic organization of phospholipid biosynthesis remain largely unexplored. The observation that Ass and Asd localize to the midcell in *H. volcanii* suggests that phospholipid biosynthesis is, at least to some extent, organized in space (Nishibori et al. 2005; Mori et al. 2016; Abdul-Halim et al. 2020). Furthermore, despite not sharing the typical membrane-integral CAPT sequence and lacking transmembrane helices, *E. coli* PssA has been shown to be associated with the membrane where it forms complexes with acyl carrier protein (ACP), PlsB and YbgC, suggesting that phospholipid biosynthesis is indeed organized in space (Gully and Bouveret 2006). With the observation in *H. volcanii*, it is not known whether these enzymes are specifically co-located, potentially forming a functional complex; or whether these proteins are associated due to an association with cell wall biosynthesis (Nishibori et al. 2005).

BLAST analysis shows that *H. volcanii* Asd is only distantly related to characterized homologs from *M. thermoautotrophicus* and *B. subtilis.* The active domains are conserved, but the evolutionary distance between these enzymes is quite large. Several motifs containing essential residues for the maturation of the enzyme have been identified (Li and Dowhan 1990; Choi et al. 2015). Interestingly, HvAsd lacks one of the conserved two histidine residues (H147 in EcPsd, R89 in HvAsd and H198 in in the Psd of the eukaryote *Plasmodium knowlesi* (PkPsd)). The latter has been shown to partake in the autoproteolytic triad and be essential for pro-peptide maturation (Choi et al. 2015). However, this particular histidine residue was not essential for the autoproteolytic processing of Psd1 in the eukaryote *Saccharomyces cerevisiae*, for which H345 was the essential residue (corresponding to H144 in EcPsd, H86 in HvAsd and H195 in PkPsd) (Ogunbona et al. 2017).

EcPsd is capable of decarboxylating archaetidylserine (AS) to form archaetidylethanolamine (AE) (Caforio et al. 2015). However, earlier studies hitherto did not provide a strong structural basis for the substrate specificity of Psd enzymes and it is uncertain how well structures are conserved between bacterial Psd and archaeal Asd enzymes. Nevertheless, it appears that the enzyme is rather promiscuous, as it can accept both bacterial and archaeal phospholipid substrates; suggesting that Psd has no, or only a mild preference for glycerol backbone chirality, or diester over diether phospholipids (Caforio et al. 2015). Also, there do not seem to be specific binding pockets for the radyl groups present in this enzyme. However, non-specific hydrophobic radyl group interactions are required for proper substrate binding and activity, as serine and phosphoserine do not compete with phosphatidylserine as substrate and glycerophosphoserine is not decarboxylated by EcPsd (Dowhan et al. 1974). Instead, the specific interactions of EcPsd are centered around the domain-agnostic PS headgroup with the predominantly positively charged active site drawing in PS and stabilizing the phosphate backbone through Y137, S166 and potentially the backbone amide of V167. Unfortunately, residue Y137 is not conserved in archaeal Asds and is replaced with phenylalanine, while S166 is replaced with a proline or valine which all lack a polar group to stabilize the phosphate headgroup. These residues are of critical importance in EcPsd; therefore, the mode of substrate stabilization in archaeal Asd remains to be elucidated (Watanabe et al. 2020; Cho et al. 2021).

#### Phosphatidylglycerol and phosphatidylinositol synthesis

A very common phospholipid headgroup found in both Bacteria and Archaea is a phospholipid with a glycerol headgroup, phosphatidylglycerol (PG) and archaetidylglycerol (AG), respectively. While phosphatidyl *myo*-inositol (PI) is not as common in Bacteria, archaetidylinositol (AI) is a relatively common phospholipid in Archaea. PG and PI are synthesized in similar ways from CDP-DAG in two steps: In Bacteria, the CMP headgroup is exchanged with G3P by phosphatidylglycerol phosphate synthase (Pgs) or with 1L-*myo*-inositol-1- phosphate^3^ by phosphatidyl inositol phosphate synthase (Pis, occasionally also referred to as Pgs); both enzymes are CAPTs, forming phosphatidylglycerol phosphate (PGP) and phosphatidyl inositol phosphate (PIP), respectively (Morii et al. 2010) (Fig. 8). The G3P and inositol phosphate headgroups of PGP and PIP are subsequently dephosphorylated by phosphatidylglycerol phosphatase (Pgp) and an unidentified phosphatidyl inositol phosphatase (Pip) to yield the plain glycerol- and inositol-headgroups of PG and PI, respectively (Morii et al. 2009; Belcher Dufrisne et al. 2020). Although it is thought that, like bacterial Pgs, the archaeal homolog (Ags) uses G3P as a substrate (Caforio et al. 2015); to our knowledge, so far, no experimental evidence has been published to confirm the stereospecificity of archaeal Ags for G1P or G3P.

**Fig. 10:**
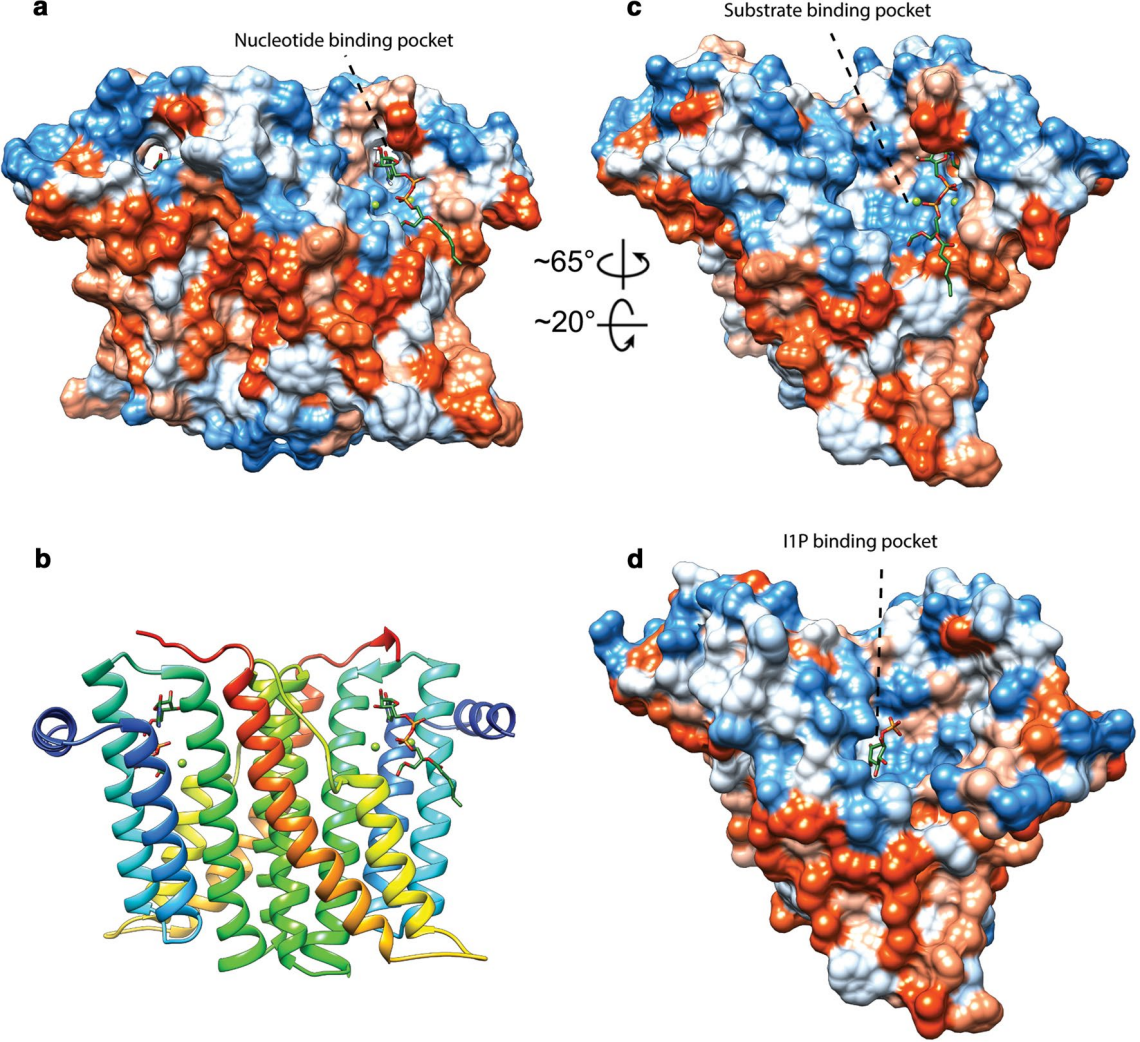
Solid-surface view (**a**, **c**, **d**) and ribbon view (**b**) of the MtPis dimer (**a**, **b**, **c**) and MkPis dimer (d) crystal structure with bound CDP-DAG and inositol-1-phosphate, respectively (PDB: 6H59 and 6WMV, (Grāve et al. 2019) and (Belcher Dufrisne et al. 2020), respectively). The surface views are colored according to the Kyte–Doolittle hydrophobicity scale where red surfaces are hydrophobic, white is of mixed character and blue is hydrophilic. Mg2 + ions are shown as green spheres

*E. coli* Pgs (PgsA) contains the hallmark CAPT-motif and is predicted to form 6 transmembrane helices forming an integral membrane protein which is typical for members of the CAPT family (Chang and Kennedy 1967; Hirabayashi et al. 1976). Only recently, the first crystal structure of a bacterial PgsA has been reported for *Staphylococcus aureus* (SaPgsA) (Yang et al. 2021). PI synthesis has been studied in more detail and three structures of bacterial Pis are deposited in PDB (Clarke et al. 2015; Grāve et al. 2019; Belcher Dufrisne et al. 2020) (Fig. 10). Several archaeal homologs of bacterial Pgs, Pis and Pgp (Ags, Ais and Agp) have been identified (Daiyasu et al. 2005; Morii et al. 2014). However, to date, no structures of archaeal Ags, Ais or AgpA have been reported, nor have these enzymes been studied in detail.

Both Pgs and PgpA from *E. coli* have been shown to be active on the archaeal analogs of CDP-DAG (CDP-archaeol) and phosphatidylglycerol phosphate (archaetidylglycerol phosphate, AGP) to form archaetidylglycerol (Caforio et al. 2015). *E. coli* contains two other Pgp homologs, PgpB and PgpC (Icho and Raetz 1983; Funk et al. 1992; Lu et al. 2011). PgpB has a distinct substrate specificity from PgpA. While PgpA only dephosphorylates PGP, PgpB is also active on DAG pyrophosphate, PA and LPA (Icho 1988; Funk et al. 1992).

The binding of inositol-1-phosphate (I1P) to Pis is dependent on the binding of CDP-DAG, suggesting an ordered sequential bi–bi reaction mechanism as is common for most other members of the CAPT family (Cleland 1963; Hirabayashi et al. 1976; Bae-Lee and Carman 1984; Dutt and Dowhan 1985). It has been suggested that the reaction mechanism involves the deprotonation of an I1P hydroxyl group by a general base which enables the subsequent nucleophilic attack of I1P on the β-phosphate of CDP-DAG. The reaction presumably proceeds through a penta-coordinated intermediate before breaking the diphosphate bond and releasing the phosphatidylinositol phosphate and CMP products (Grāve et al. 2019). The activity of bacterial Pis on archaeal substrates compared to bacterial substrates is considerably lower, indicating some degree of substrate specificity (Morii et al. 2014). Remarkably, the same study shows that while A is accepted both substrates, it showed more activity on bacterial substrates compared to archaeal substrates. The latter may also be caused by non-optimal reaction conditions, as previously, it was shown that Ais activity in *M. thermoautotrophicus* membranes was greatly stimulated by increasing the detergent concentration (Morii et al. 2009). Variations in used detergent concentration may give rise to activity differences.

The crystal structures of Pis from *Mycobacterium tuberculosis* (MtPis) and *Renibacterium salmoninarum* show these enzymes as dimers, possessing the typical CAPT structure with 6 transmembrane helices per monomer forming integral membrane proteins (Clarke et al. 2015; Grāve et al. 2019) (Fig. 10). The MtPis crystal structure notably reveals the binding mode of two catalytically important Mg^2+^ cations which are coordinated by D68, D71, D89 and D93 of the conserved CAPT-motif (Fig. 10b, c). Due to the complete loss of activity of D93 mutants, it has been suggested that, in addition to coordinating one of the Mg^2+^ ions, D93 likely acts as a catalytic base for the deprotonation of the I1P hydroxyl group (Aktas et al. 2014; Nogly et al. 2014; Clarke et al. 2015; Grāve et al. 2019). The cytidyl nucleotide moiety of CDP-DAG is bound into a pocket between TM helices α1-α3 (Fig. 10a, c). The nucleotide-binding pocket is lined by G72, A75, G85 and residues D31, T34 and T82 form weak hydrogen bonds with the nucleotide moiety. The diphosphate moiety of CDP-DAG interacts with at least one of the Mg^2+^ ions and the α-phosphate is additionally coordinated by G72 and R76. MtPis possesses a pronounced hydrophobic cleft running from the negatively charged diphosphate binding site containing the Mg^2+^ ions on the intracellular side of the enzyme toward the extracellular side of the enzyme (Clarke et al. 2015). Although the CDP-DAG radyl tails were poorly resolved and therefore truncated in the crystal structure, this hydrophobic cleft is presumed to be the radyl group-binding site. The crystal structure of engineered *Mycobacterium kansasii* Pis (MkPis) shows the same general features but also shows the binding site of the phosphatidyl acceptor substrate I1P (residues are numbered according to MtPis) (Belcher Dufrisne et al. 2020) (Fig. 10d). Residues S132, K135, R155 and R195 were previously hypothesized to be involved in I1P binding and the MkPis crystal structure confirms this. Residue R94 and residues Y133 and R137 of the other peptide chain are positioned to interact with the phosphate moiety of I1P. Mutagenesis of R137 dramatically reduces enzyme activity, indicating that the enzyme is functional as a dimer. Residue R152 is structurally strictly conserved in CAPT enzymes and is positioned to form the floor between the I1P and CDP-DAG-binding pockets. Despite its essentiality, the function of R152 remains to be elucidated.

The structure of SaPgsA appears highly reminiscent of the MtPis crystal structure described above (Grāve et al. 2019; Yang et al. 2021). The phosphatidyl moiety of the co-crystallized PGP and CDP-DAG moieties is positioned in virtually the same way. Moreover, the cytidyl nucleotide headgroup of CDP-DAG is bound to the CDP-binding pocket and oriented in the same way compared to the CDP-DAG in the MtPis crystal structure. Finally, the phosphatidyl acceptor-binding site of SaPgsA (G3P) seems somewhat conserved. Mainly lysine and arginine residues that are located on similar positions as I1P-binding residues in MtPis interact with the G3P polar headgroup of co-crystallized PGP.

Overall, Archaea and Bacteria use similar types of enzymes to produce the various phospholipids with common polar headgroups. These enzymes likely have a common origin which was possibly already present in the last universal common ancestor (LUCA) (Koga 2011, 2014; Lombard et al. 2012b). So far, several studies showed that CAPTs can show some specificity, but ultimately tend to accept both archaeal and bacterial substrates (Morii and Koga 2003; Morii et al. 2014; Caforio et al. 2015). However, CAPT substrate preference has not been studied in detail, while these enzymes are expected to be more likely to show some substrate specificity between archaeal and bacterial substrates when compared to downstream enzymes. CAPTs act on the shared CDP headgroup, they are integral membrane proteins and thus interact more closely with the lipid radyl groups of phospholipid substrates compared to downstream enzymes (Sciara et al. 2014; Nogly et al. 2014; Grāve et al. 2019). Because of the diversity of downstream enzymes and limited structural insights, aspects of enzyme substrate specificity remain to be elucidated.

#### Cardiolipin synthesis

Cardiolipins (CL) are a class of phospholipids present in membranes in all three domains of life and were found to be involved in osmoregulation, membrane organization and is associated with bioenergetic proteins (Schlame and Greenberg 1997; Corcelli et al. 2000; Haines and Dencher 2002; Lobasso et al. 2003; Lopalco et al. 2004; Bogdanov et al. 2008; Schlame 2008; Romantsov et al. 2009, 2018; Schlame and Ren 2009; Klingenberg 2009; Tsai et al. 2011; Arias-Cartin et al. 2012; Mühleip et al. 2019). The structure of CL is rather unusual as it involves a phospholipid linked to a phospholipid or glycolipid through a polyol headgroup such as glycerol or a sugar moiety. The most commonly studied cardiolipin is 1,3-bis(sn-3’-phosphatidyl)-sn-glycerol, referred to as glycerol-di-phosphatidyl cardiolipin (Gro-DPCL) or simply cardiolipin. This phospholipid is usually a relatively minor component of the lipidome of *E. coli* or *B. subtilis* and has also been identified in Archaea (Corcelli et al. 2000; Lattanzio et al. 2002; Sprott et al. 2003; Lobasso et al. 2003; Yoshinaga et al. 2012; Angelini et al. 2012; Bale et al. 2019). Notably, one of the cardiolipin species identified in a halophilic archaeon, was a sulfated glyco-cardiolipin, found strongly associated with bacteriorhodopsin (Corcelli et al. 2000). The headgroup of the sulfated glyco-cardiolipin bears a resemblance to phosphorylated- or sulfated hexoses such as di-*myo*-inositol phosphate or trehalose-sulfate which were both identified in archaea as osmolytes (Desmarais et al. 1997; Chen et al. 1998; Roberts 2004). Moreover, the stimulation of de novo synthesis of these lipids upon osmotic shock suggests that these lipids could play a role in osmoadaptation and stabilization of proteins (Lobasso et al. 2003; Lopalco et al. 2004; Corcelli 2009).

Two main types of Cls have been identified. Typically, bacterial cardiolipin synthases (Cls) are members of the phospholipase-D (PLD) superfamily which also contains PS synthases (EcPssA), several endonucleases, poxvirus envelope proteins, a murine toxin from *Yersinia pestis* and the prototypical phospholipase-D enzymes after which the superfamily is named (Sandoval-Calderón et al. 2009). PLD-type Cls synthesize CL through the reversible condensation of two phosphatidylglycerol moieties, forming CL and releasing a glycerol moiety in the process. In addition to cardiolipin synthase activity, PLD-type Cls have been shown to exhibit typical phospholipase-D activity as well, irreversibly hydrolyzing the headgroup of PG to yield PA and glycerol (Jeucken et al. 2018; Exterkate et al. 2021). The second group of Cls belongs to the CAPT family and has been identified in eukaryotes; forming CL by linking CDP-DAG to the terminal hydroxyl group of the glycerol headgroup on PG, releasing a CMP moiety in the process (Schlame and Greenberg 1997; Sandoval-Calderón et al. 2009). CAPT-type Cls have only been found only in Eukaryotes and Actinobacteria. Hence, this work will only discuss PLD-type Cls from this point onward as these are also present in Archaea.

**Fig. 11:**
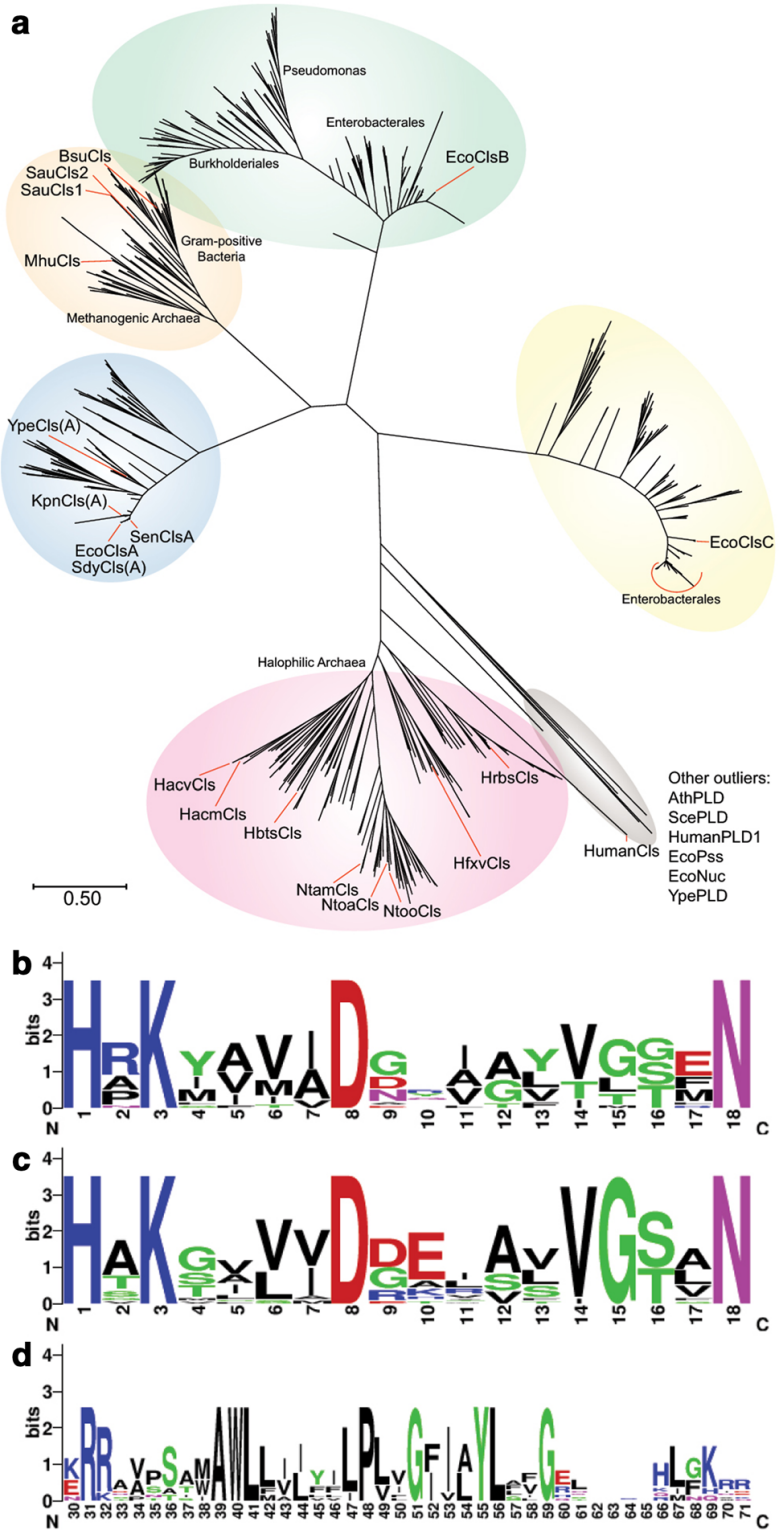
(**a**) A maximum-likelihood phylogenetic tree showing the phylogenetic distribution of different types of Cls. Weblogo sequence of the first (**b**) and second HKD motif (**c**) from a selection of in total 18 Cls from well-known bacteria, MhCls and several haloarchaeal PLD-type Cls homologs (Crooks et al. 2004). The weblogo of the second N-terminal hydrophobic region (**d**) was generated from only bacterial ClsA-type enzymes and MhCls. The phylogenetic tree was constructed from sequences obtained by BLAST analysis on archaea and bacteria with a maximum of 5000 hits using EcClsA, B, C, MhCls and Cls homologs from H. volcanii and Halobacterium salinarum. Prior to maximum-likelihood phylogenetic tree construction using the JTT model (Jones et al. 1992) in MEGA-X (Kumar et al. 2018), EcClsA, B and C hit redundancy was reduced to 80% sequence similarity and for the other queries 99%. The top-200 hits of each redundancy reduced query were then pooled, duplicates removed, and redundancy was again reduced to 80% sequence similarity which yielded a final list of 798 hits. Cls homologs (18) of selected organisms and outliers (7) were added before alignment using Clustal Omega (Madeira et al. 2019)

PLD-type Cls have been subdivided into 4 subtypes based on various properties: ClsA-, ClsB- and ClsC-type and putative halophilic archaeal Cls. ClsA-types contain a conserved hydrophobic N-terminal domain not found in ClsB or ClsC (Guo and Tropp 2000). ClsC clearly forms a different group on the basis of sequence similarity to ClsA and ClsB and EcClsC has been shown to be capable of utilizing PE in contrast to EcClsA and EcClsB (Tan et al. 2012). The putative haloarchaeal Cls form a separate phylogenetic group, distinct from ClsA, ClsB, ClsC and known PLDs (Exterkate et al. 2021) (Fig. 11a). Interestingly, phylogenetic analysis revealed at least 5 fairly distinct groups. Based on the phylogenetic tree, one could argue that the ClsA group could be subdivided into ClsA1 and ClsA2; with both groups containing members which possess the conserved N-terminal region and ClsA1 mostly containing members from Gram-negative bacteria and the ClsA2 group methanogenic archaea and Gram-positive bacteria (Fig. 11a).

Some Bacteria possess multiple Cls homologs. *Staphylococcus aureus* is known to possess at least two closely related Cls homologues, of which one is thought to be active during low-pH stress conditions in which the other Cls is not active (Ohniwa et al. 2013). *E. coli* possess three cardiolipin synthases, ClsA, ClsB and ClsC, of which ClsA has been the most thoroughly studied (Guo and Tropp 2000; Tan et al. 2012). Archaea typically contain a single Cls homolog, but their distribution is mostly restricted to halophiles and methanogens. In *E. coli,* the expression of the Cls homologues differs during the various stages of culture growth and stress conditions. The exact purpose of multiple Cls is not completely clear. Aside from differential expression during different growth phases or stress conditions, different Cls subtypes have a different substrate specificity allowing them to either use different phospholipid and/or alcohol substrates (Tan et al. 2012; Li et al. 2016; Reinink et al. 2019). However, this has not been exhaustively studied with comparable in vitro conditions for all different Cls subtypes from a single organism or close homologs. Perhaps, Cls enzymes are involved in phospholipid remodeling.

PLD-type Cls, like typical PLD enzymes, contain a duplicated PLD domain which consists of a set of four structural regions. The third region of the set contains a highly conserved HKD motif (H–x-K-x(4)-D) for a total of two HKD motifs per Cls enzyme (Koonin 1996; Ponting and Kerr 1996). Some sources cite the HKD motif to be longer, including residues from the fourth structural region (H–x-K-x(4)-D-x(6)-G-[GST]-x-N), while others refer to these extra residues as a separate motif (Sung et al. 1997; Stuckey and Dixon 1999; Guo and Tropp 2000). Notably, the degree of conservation between the first and second (extended) HDK motif among Cls is different. With the Cls from several well-known Bacteria, including the Cls from *Methanospirillum hungatei* (MhCls) and several putative haloarchaeal PLD-type Cls, the first extended HKD motif is less conserved and rather resembles “H–x-K-x(4)-D-x(6)-[GLT]-[GST]-x-N” whereas the second HKD motif is more defined and resembles the cited motif “H–x-K-x(4)-D-x(5)-[VI]-G-[ST]-x-N” (Fig. 11b, c). Additionally, *E. coli* ClsA (EcClsA) contains an N-terminal domain of approximately 60–141 residues in length which seems to be present in ClsA homologs from other bacteria (and including MhCls). It is especially prevalent in Gram-negative bacteria such as members of the Enterobacterales (Quigley and Tropp 2009). The regions of residues 7–29 and 33–64 in the N-terminal domain of EcClsA contain stretches of hydrophobic residues flanked C-terminally by positively charged residues and are predicted to each form a transmembrane helix. The second hydrophobic region contains a motif of unknown function and is conserved among ClsA of some well-known bacteria and MhCls (Exterkate et al. 2021) (Fig. 11d). Protein mass fingerprinting and Edman degradation data suggest that EcClsA undergoes a post-translational modification at its N-terminus resulting in the loss of a region from residue 1 up to residue 32 that harbors the first hydrophobic region (unpublished data) (Ragolia and Tropp 1994; Romantsov et al. 2018)).

Previous data indicated that ClsA from *E. coli* accepts substrates other than glycerol for the reverse “alcoholysis” reaction of CL, such as simple primary alcohols and D-mannitol (Shibuya et al. 1985; Jeucken et al. 2018). The ClsA-type homolog from the methanogenic archaeon *M. hungatei* (MhCls) has been characterized and shown to exhibit a high degree of promiscuity. In contrast to the EcClsA, the enzyme does not seem to undergo the same post-translational modification and could be purified as a full-length protein. It was found to accept various alcohols other than glycerol in the reverse reaction. This resulted in the synthesis of natural and various non-natural phospholipids in vitro. Furthermore, MhCls was found to be active on bacterial G3P-based ester phospholipids in addition to the archaeal G1P-based ether phospholipids (Exterkate et al. 2021). Remarkably, the enzyme is capable of generating a hybrid phospholipid species, not before seen in nature, where PG is coupled to AG. Notably, both EcClsA and MhCls are unable to utilize trehalose as an alcohol acceptor (unpublished results) unlike the S*almonella typhi* ClsB that is responsible for the formation of phosphatidyl trehalose and di-phosphatidyl trehalose cardiolipin (Reinink et al. 2019).

**Fig. 12:**
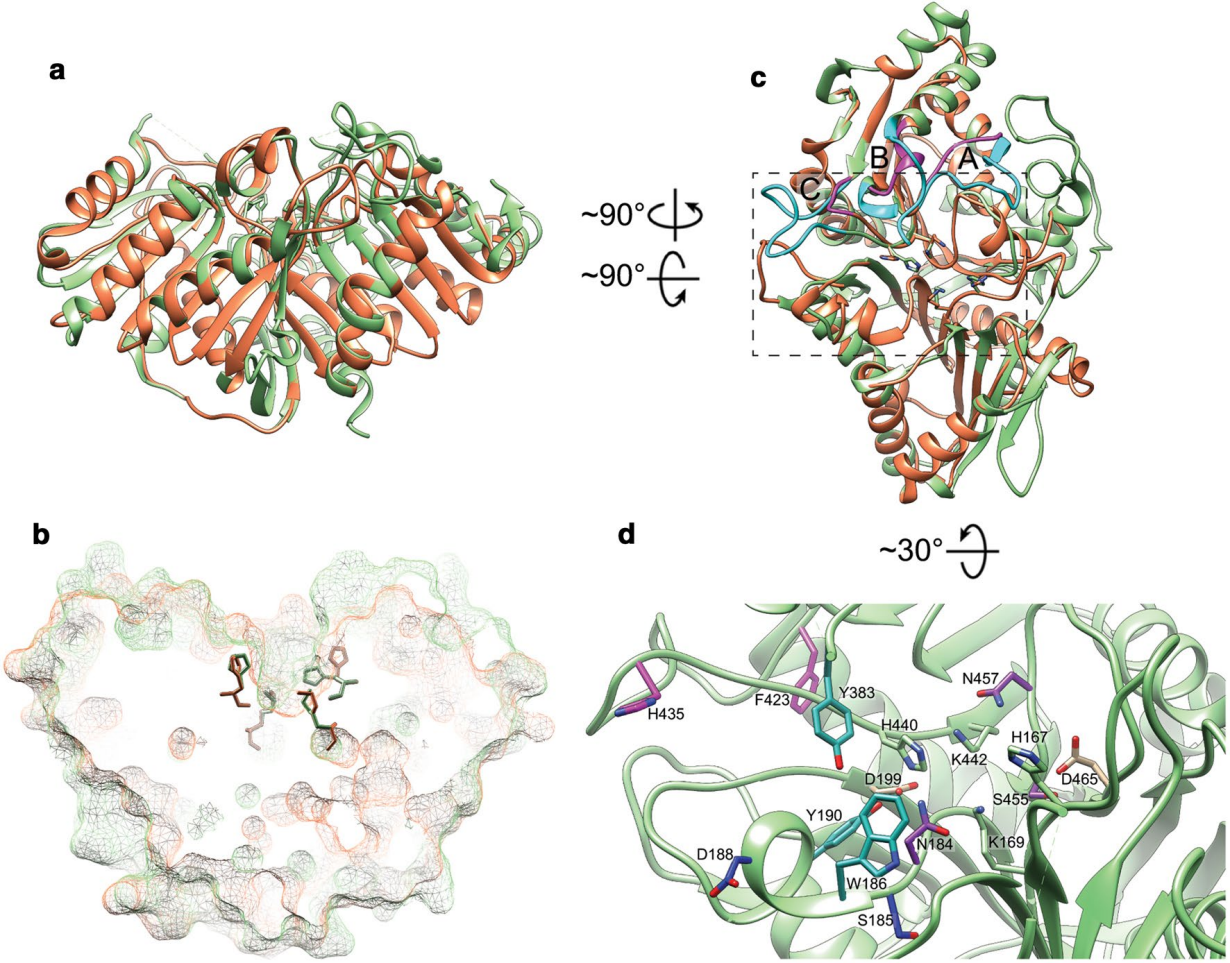
Ribbon view (**a**, **c**, **d**) and mesh-surface view (**d**) of the PMFPLD crystal structure (PDB: 1F0I, (Leiros et al. 2000)). (**a**, **c**) The EcClsA structure (orange) is modeled onto the PMFPLD crystal structure (light green) where loops A, B, and C are colored magenta and cyan for the EcClsA model and PMFPLD structure, respectively. (**b**) Mesh surface section of the EcClsA model and PMFPLD structure with the histidine and lysine residues of the HKD motifs shown as sticks. (**d**) A tilted and zoomed-in view of panel c (visible area marked) in which histidines and lysines of the HKD motif and residues discussed in the text are shown as sticks and are colored according to cited studies in which they, or corresponding residues in other PLDs are mutated (blue (Uesugi et al. 2005), magenta (Uesugi et al. 2006), and dark-cyan (Masayama et al. 2008)). Or purple for residues of interest other than glycine in the extended HKD motif (Sung et al. 1997; Ogino et al. 2007), or tan for two conserved aspartic acid residues involved in catalysis

No PLD-type Cls crystal structures have been published to date. However, crystal structures of other PLD superfamily members from eukaryal and bacterial sources have been reported (Stuckey and Dixon 1999; Leiros et al. 2000; Li et al. 2020). The EcClsA structure has been modeled based on a bacterial PLD family member structure using the Phyre2 one-to-one threading function, resulting in a saddle- or bilobed structure typical for PLD structures (Leiros et al. 2000; Kelley et al. 2015; Romantsov et al. 2018; Li et al. 2020) (Fig. 12a). The modeled EcClsA structure lacks a pronounced hydrophobic surface, and thus, the mechanism of membrane association remains unclear if in the native enzyme both predicted N-terminal transmembrane helices are indeed cleaved off (Quigley and Tropp 2009). The conserved histidine and lysine from both HKD motifs are located in close proximity of one another in a pocket at the interface of the PLD domain lobes (Fig. 12b, c). The catalytic pocket is further lined with the glycine (G238, G418), serine (S239), threonine (T419), asparagine (N241, N421) residues of the extended HKD motif, and seems to be larger than the binding pocket of the template PLD (*Streptomyce*s sp. PMF, PMFPLD, PDB: 1F0I, (Leiros et al. 2000)). Although, most of the difference in the size of the catalytic pocket seems to be caused by the “external” conformation of a HKD motif histidine (H448) in the PMFPLD structure compared to the EcClsA model where both HKD motif histidines are in the “internal” conformation and thus can be attributed to a modeling- or crystallization artifact (Leiros et al. 2000, 2004) (Fig. 12b). The PMFPLD crystal structure shows three loops (Loop A, B and C) near the catalytic pocket which could be functionally significant, potentially inhibiting the binding of larger phosphatidyl acceptor substrates compared to EcClsA (Fig. 12c). Loop A, B and C in PMFPLD are formed from three sequences inserted between predicted structurally conserved elements in a region between structural region II and III exclusively in the C-terminal PLD domain (D336-R350, A373-L389 and F423-Y437 in PMFPLD corresponding to Y343-D349, R370-L376 and F398-G401 in EcClsA resp). Particularly loop B in PMFPLD (A373-L389) is situated near the C-terminal histidine residue which activates the acceptor alcohol. Loop B might provide a structural basis for PMFPLDs preference for phosphodiester hydrolysis instead of transphosphatidylation by reducing space in- or access to the active site. However, as models only have limited accuracy, the orientation of residues might be different in an actual crystal structure of EcClsA and enzymatic conformation could be different in an aquatic environment or in presence of a phospholipid bilayer. Moreover, PLDs are thought to undergo some conformational shifting around the active site upon the binding of substrates or inhibitors, and thus, no strong conclusions can be drawn from this modeled result (Leiros et al. 2004; Ogino et al. 2007; Li et al. 2020).

Even though the (extended) HKD motif is conserved among PLD family members, they show different substrate and/or product specificities and different ratios of hydrolysis to transphosphatidylation (Hagishita et al. 2000; Sato et al. 2004; Nakazawa et al. 2009). The latter of which can also be interpreted as a difference in substrate specificity of water versus an acceptor alcohol. These observations indicate that other residues are likely responsible for substrate specificity. However, despite several solved crystal structures of PLD members, the structural basis for their substrate specificity is not known.

Several studies have attempted to clarify the structural basis of PLD-member substrate specificity. It was shown that a substitution of serine to threonine, or glycine to serine, in the “GG/GS” motif (considered to be part of the extended HKD motif) in the C-terminal structural region 4 of PLD, strongly reduced overall activity or resulted in a significant increase of transphosphatidylase activity respectively (Sung et al. 1997; Ogino et al. 2007). Notably, Cls often have a threonine instead of a serine in that position, as well. Mutational analysis of various PLDs in a region near the active pocket (Uesugi et al. 2005) or in loop C revealed residues that are reported to impact phospholipid affinity, enzyme stability, substrate specificity and transphosphatidylase or hydrolase activity (Uesugi et al. 2005, 2006; Masayama et al. 2008) (Fig. 12d). Importantly, many of these mutated residues are not conserved among PLDs. And even though the mutations of residues in loop C significantly alter the characteristics of the enzyme, the exact role of the mutated residues and loop C itself remains to be determined. Overall, while several residues affecting substrate specificity were identified in PLDs, no study has provided overarching conclusive answers.

The catalytic mechanism of PLD enzymes is conserved among the entire superfamily. All reactions involve the transesterification or hydrolysis of a glycerophosphodiester lipid with either an organic molecule containing a hydroxyl group or water, respectively. The first step of the reaction involves the formation of a phosphatidyl-enzyme intermediate through a nucleophilic attack on the phosphatidyl phosphorus atom by the histidine from the N-terminal HKD motif which is activated by a nearby conserved aspartic or glutamic acid from the C-terminal PLD domain (H224/E432 in EcClsA or H167/D465 in PMFPLD numbering, respectively (Fig. 12d)) (Gottlin et al. 1998; Leiros et al. 2000; Uesugi et al. 2005). In the second step, the histidine from the C-terminal HKD motif which is activated by an aspartic acid residue from the N-terminal PLD domain protonates the headgroup of the substrate, forming the alcohol leaving group. In the third and final step, the histidine of the C-terminal HKD motif then activates a primary alcohol or water molecule for a nucleophilic attack on the phosphatidyl-histidine intermediate, forming the free phospholipid with the new alcohol headgroup or PA, respectively.

In accordance with the catalytic mechanism discussed above, residues around the C-terminal HKD motif histidine have been shown to affect acceptor alcohol substrate specificity. Therefore, it is likely that residues around the N-terminal HKD motif histidine could influence phosphatidyl donor substrate specificity. Ultimately, enzymes of the PLD family could be more efficiently designed to reduce hydrolysis and accept larger or different alcohol substrates when cardiolipin synthases are used as design template to learn more about substrate selectivity mechanisms in PLD family members. Alternatively, Cls could be engineered to accept new donor or acceptor substrates to generate synthetic phospholipids with customized properties. A cardiolipin synthase crystal structure would further aid this research and possibly further clarify alcohol acceptor substrate recognition.

### Lipid core modification

#### Isoprene saturation

In most archaea, the isoprene chains of mature membrane glycerophospholipids are fully saturated. Saturation is assumed to occur after polar headgroup activation due to the specificity of CarS for unsaturated archaetidic acid (DGGGP) (Morii et al. 2000). Prenyl reductases are flavoenzymes responsible for the saturation of isoprenyl groups. Specifically, in the context of archaeal phospholipid biosynthesis, geranylgeranyl reductase (GGR) fulfills this function. The search for prenyl reductases in Archaea led to the identification of a membrane-associated GGR from *T. acidophilum* (TaGGR) which is active on DGGGP and partially unsaturated archaeols (di-phytyl) with a polar head group such as AE and AG (Nishimura and Eguchi 2006, 2007; Xu et al. 2010). The degree of saturation of bacterial fatty acyl chains is determined during fatty acid synthesis by FabA (anaerobic) or fatty acids are aerobically desaturated by desaturases (Cybulski et al. 2002). For example in *E. coli*, unsaturated bonds are anaerobically introduced and cis-isomerized in fatty acid synthesis intermediates by FabA and directed back into the main fatty acid synthesis cycle by FabB (Heath and Rock 1996; Fujita et al. 2007; Feng and Cronan 2009; Dodge et al. 2019). As part of the fatty acid synthesis cycle, FabZ introduces unsaturated bonds as well; however, these are subsequently saturated by FabI (Heath and Rock 1996). The saturation of fatty acid biosynthesis intermediates by FabI and introduction of unsaturated bonds by FabA (and FabZ) or aerobic desaturases occur through entirely different mechanisms compared to archaeal isoprenyl synthesis and isoprenyl saturation. Hence, bacterial fatty acid saturation is not further discussed in this work.

**Fig. 13:**
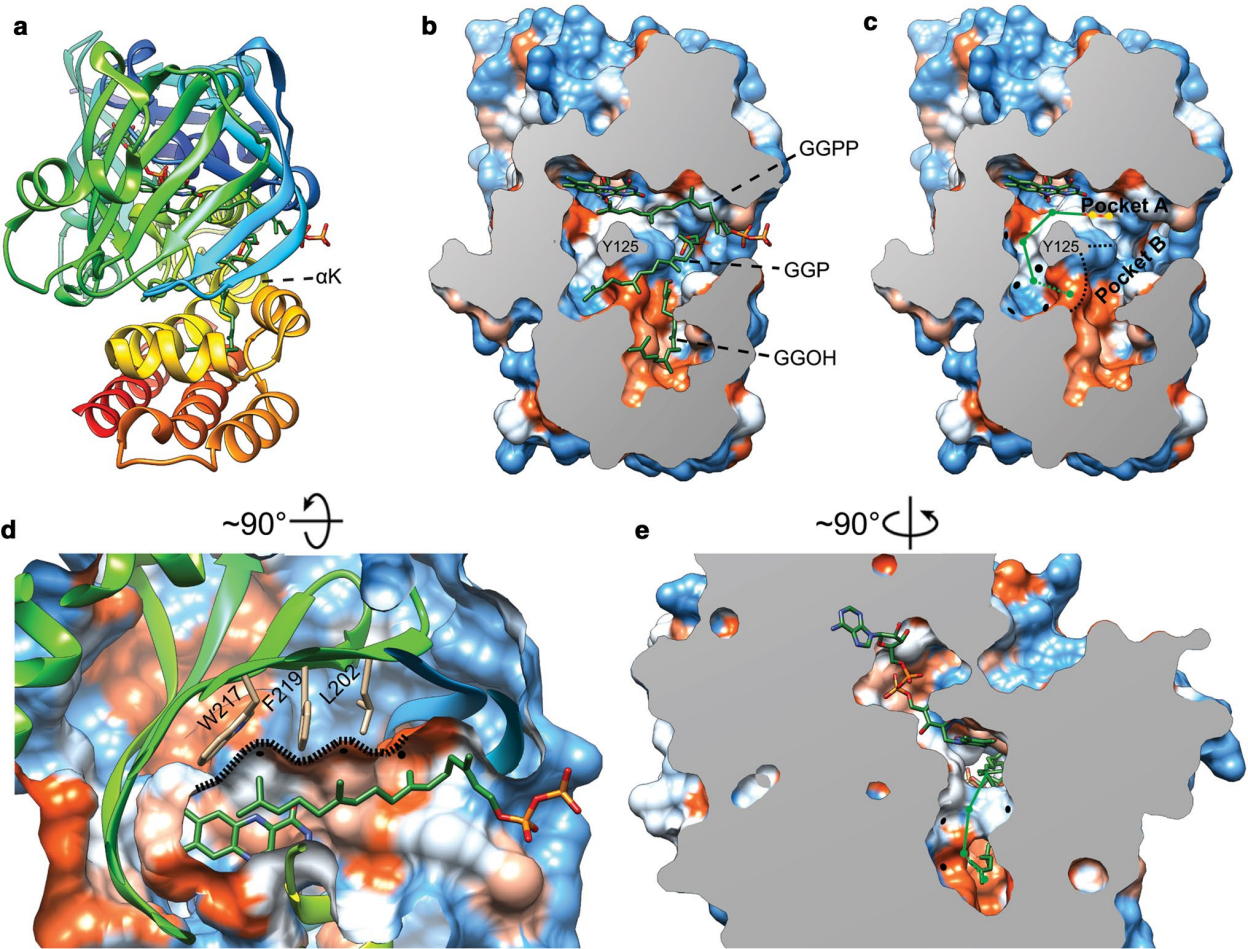
Ribbon view (**a**, **d**) and solid-surface view (**b**, **c**, **d**, **e**) of the SaGGR crystal structure with bound FAD, GGPP and its derivatives (PDB: 4OPD, (Kung et al. 2014)). The surface views are colored according to the Kyte–Doolittle hydrophobicity scale where red surfaces are hydrophobic, white is of mixed character and blue is hydrophilic. Possible positions of tri-substituted carbon atoms and the distance between them for each isoprenyl moiety in GGPP are schematically illustrated in a 2D-plane as circles and lines, respectively, with colors representing different elements (**c**, **e**). Pockets which could potentially harbor (**c**, **e**), or have been shown to be able to harbor isoprene-methyl moieties are marked with black dots (**d**)

The only two crystal structures of archaeal GGRs are those of *Sulfolobus acidocaldarius* GGR (SaGGR) and TaGGR (Xu et al. 2010; Sasaki et al. 2011; Kung et al. 2014). SaGGR is a monomer composed of two functional domains resembling TaGGR: an FAD-binding Rossman-type fold domain and a ligand-binding domain (Fig. 13a). The substrate-binding cavity is situated between the two established functional domains and contains a catalytic- (Pocket A) as well as a non-catalytic (Pocket B) hydrophobic-binding pocket to accommodate substrates containing two lipid tails (Fig. 13b, c). Interestingly, despite the high similarity between the SaGGR and TaGGR sequences and structures, SaGGR is purified from the cytosolic fraction, whereas TaGGR is associated with the cell membrane fraction. Compared to TaGGR, SaGGR contains 60 extra amino acids at the C-terminus forming three α-helices that are considered part of the ligand-binding domain. A DALI search revealed similarities with members of the *p*-hydroxy-benzoate hydroxylase (PHBH) flavin monooxygenase family which adopt a similar two-domain organization and structure, but lacking the 60 C-terminal amino acids found in SaGGR (Holm and Sander 1995; Xu et al. 2010; Sasaki et al. 2011). Notably, the PHBH family is known to have members with three-domain structures that are approximately 50–70 amino acids longer than the two-domain enzyme sequences (See (Sasaki et al. 2011) and references therein). However, the three-domain PHBH sequences were not reported to correspond more closely to SaGGR or its C-terminal sequence.

The secondary structures of the FAD-binding and ligand-binding domains are highly conserved between PHBH and GGR with some noteworthy exceptions: Helix αK at the domain interface leading into the C-terminus of the ligand-binding domain is kinked, tilting the C-terminal portion of the ligand-binding domain away from the substrate-binding pocket opening and attributing to the creation of a larger internal cavity compared to PHBH enzymes (Xu et al. 2010) (Fig. 13a). This larger cavity accommodates the lipid tail which is bound in the non-catalytic pocket (Pocket B) of a diether phospholipid substrate such as DGGGP.

The two hydrophobic-binding pockets for the accommodation of substrate lipid tails are separated by Y215 (SaGGR numbering) and a loop from residues 290–301 with Y215 forming a hydrogen bond with the backbone carbonyl of H297 (Kung et al. 2014) (Fig. 13b, c). While co-crystallized PG from the expression host in the TaGGR structure did not form many specific interactions, the phosphate headgroup moiety of PG in the SaGGR crystal structure formed salt-bridges with residues H55, H297 and hydrogen bonds with residues Y340 and N294. This is remarkable as the PG from the expression host has a G3P-based backbone, whereas archaeal phospholipids as found in *S. acidocaldarius* have a G1P-based backbone. This indicates that SaGGR might be glycerol backbone agnostic, despite having fairly deep substrate-binding pockets as the different chiral center of G3P vs G1P only affects the positioning of the secondary lipid tail in the non-catalytic position. However, this would depend on whether the bulkier isoprenyl-ether substrate can also fit in pocket B without hindering the positioning of the primary isoprenyl lipid tail in the catalytic pocket when bound to a G3P-based backbone.

When co-crystallized with GGPP, three moieties corresponding to GGPP with different phosphorylation states were found in both hydrophobic-binding pockets of SaGGR (Kung et al. 2014) (Fig. 13b). Only one GGPP moiety was bound in a catalytic manner (1, GGPP) in pocket A, while the other two moieties were found in pocket B (2, GGP and 3, GG-OH). The GGPP phosphate moiety was found to bind in a different manner compared to the bacterial PG in TaGGR or SaGGR and only forming a hydrogen bond with the β-phosphate to the backbone carbonyl of N90 (Xu et al. 2010; Kung et al. 2014). The terminal double bond of GGPP (∆^14^) is in a suitable position for reduction, directly facing the N-5 nitrogen of FAD (Fig. 13b, d). Notably, the methyl group directly adjacent to this double bond is situated between two conserved residues (W217 and F219) which likely act as a biological resistive detent mechanism or stabilization feature to position the double bond properly for catalysis in concert with Y215 (Kung et al. 2014) (Fig. 13d). Moreover, particularly pocket A seems to contain more sub-pockets that occur with a spacing that might be suitable to accommodate isoprene-methyl moieties, suggesting that these pockets could harbor the lipid methyl groups for when the isoprenoid substrate is bound differently for the reduction of double bonds other than the terminal bond (Fig. 13c, d, e). While the GGP moiety is bound in a non-catalytic manner, its phosphate headgroup engages in similar interactions to PG and forms salt-bridges with H55, H297 and additionally K343, and hydrogen bonds with Y340 and N294; indicating that these indeed play a role in binding anionic moieties such as the phosphate moiety in glycerophospholipids such as GGPP, GGGP, DGGGP, unsaturated CDP-archaeol or headgroup modified derivatives thereof (Kung et al. 2014; Cervinka et al. 2021).

Sato et al. (Murakami et al. 2007; Sato et al. 2008) showed that purified GGR from *S. acidocaldarius* (SaGGR) was more active than a GGR isolated from *A. fulgidus* (AfGGR). Interestingly, it was also observed that SaGGR exhibited a different reduction pattern depending on the substrate, fully reducing diether-committed geranylgeranyl moieties such as GGGP and only partially reducing GGPP, leaving the proximal double bond intact and forming Phytyl-PP (Sato et al. 2008). In recent years, two studies have shown that GGRs have a surprisingly broad substrate specificity, as in addition to GGPP, GGGP and DGGGP, AfGGR and SaGGR are also active on terpene acids, free terpene alcohols and terpene alcohol derivatives with headgroups other than pyrophosphate, such as hemiester, ester and aromatic headgroups (Meadows et al. 2018; Cervinka et al. 2021). In contrast to earlier observations, SaGGR was shown to be able to fully reduce a portion of both GGPP, geranylgeraniol and farnesol substrates (Sato et al. 2008; Meadows et al. 2018; Cervinka et al. 2021). It should be stressed that full reduction of GGPP, forms phytanyl-PP, which is no longer suitable as a prenyl donor and therefore a dead-end-product. Saturation of the proximal double bond prevents stabilization of the carbocation intermediate, as seen in GGPP during catalysis by GGGPS, IPPS and likely DGGGPS (Zhang and Poulter 1993; Blank et al. 2020). The activity of GGR on terpene acids is remarkable, as this indicates that the carbonyl oxygen of bacterial acyl chains should not prevent substrate recognition by GGR. However, unsaturated acyl chains typically lack the methyl groups of isoprene chains which could potentially prevent efficient catalysis as these methyl groups are thought to play a role in substrate stabilization in the active site as seen with GGPP (Kung et al. 2014). Both AfGGR and SaGGR were found to saturate synthetic isoprenoids with a succinate ester headgroup closer to completion compared to free isoprenols or isoprenoids with an acetate ester or benzyl ether headgroup, suggesting that the distance between charged (or polar) headgroup atoms to the proximal double bond is important for reduction of (or the lack thereof) the proximal bond (Cervinka et al. 2021). By extension indicating that the difference in product specificity between AfGGR and SaGGR is caused by a longer distance from the active site to the phosphate-binding site in SaGGR.

The native biological reducing agent for some GGRs remains elusive. The activity of identified GGRs in vitro is dependent on the abiotic reducing agent sodium dithionite; although activity of TaGGR has been observed in the presence of NADH and a low level of activity of SaGGR was observed with extremely high concentrations of NADH (Nishimura and Eguchi 2006; Sasaki et al. 2011). SaGGR and GGR from *Methanosarcina acetivorans* (MaGGR) are only poorly active when heterologously expressed in *E. coli*, suggesting either folding issues or that the non-covalently bound FAD co-factor of GGR cannot be effectively reduced in preparation for catalysis (Isobe et al. 2014). By introducing a ferredoxin-like protein encoded downstream to the GGR gene in the *M. acetivorans* genome, increased GGR activity was observed in *E. coli* cells (Isobe et al. 2014). A SyntTax synteny conservation search revealed that the GGR-ferredoxin synteny is largely conserved among selected Euryarchaeotes with the exception of members of the Halobacteriales and notably, *A. fulgidus* and *M. mazei* (Oberto 2013). However, the GGR-ferredoxin synteny found in *M. acetivorans* is not conserved in any Crenarchaeote in the SyntTax database (unpublished results).

### Glycerol dialkyl glycerol tetraether (GDGT) biosynthesis and polar headgroup modification

**Fig. 14:**
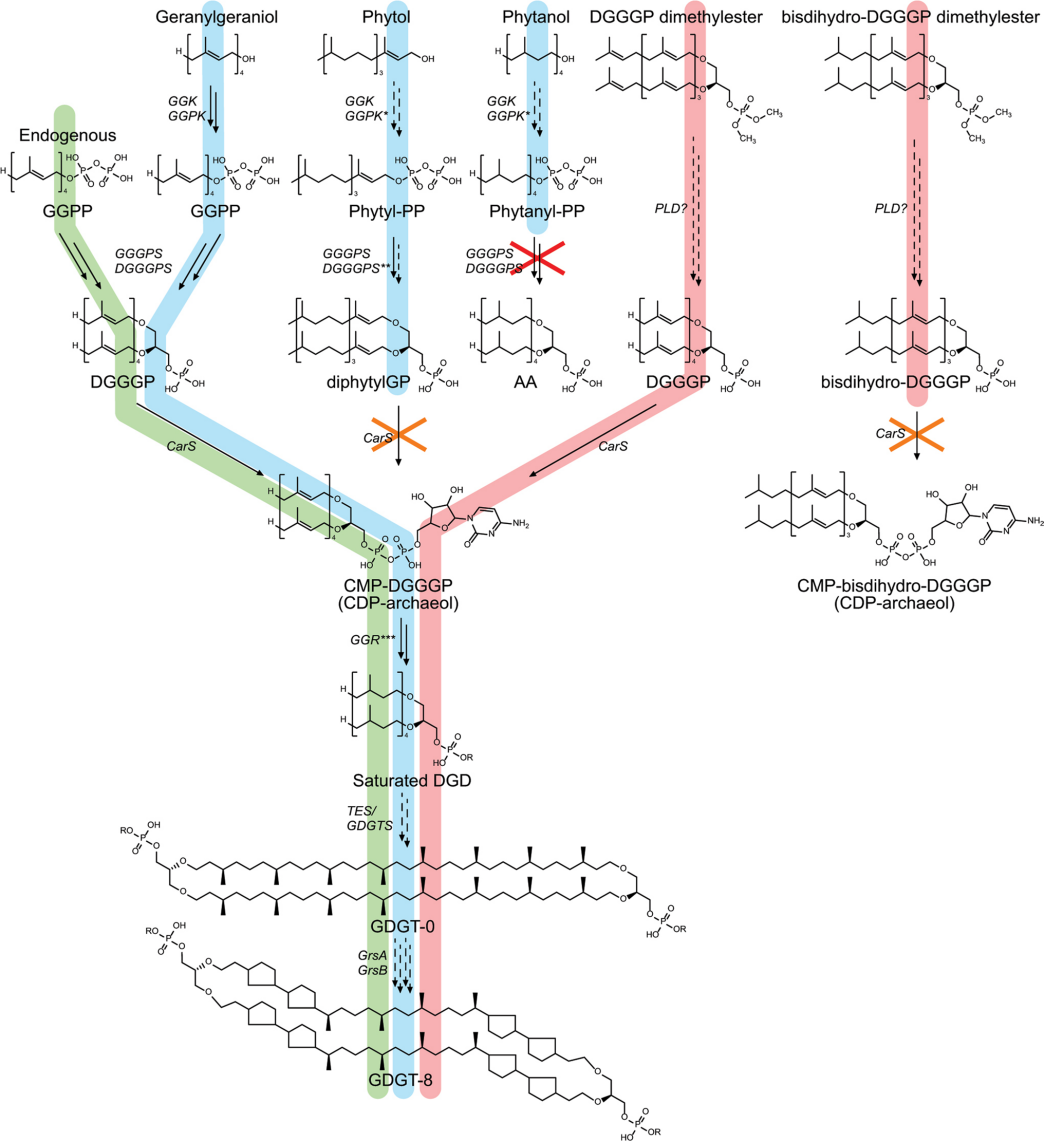
Proposed biosynthetic pathway with GGGPS, DGGGPS and CarS acting as (isotopically labeled) feeding substrate gatekeepers. The pathway for endogenous GGPP is colored green, while the experimental feeding substrate pathways are indicated in blue (Poulter et al. 1980) and red (Eguchi et al. 2003). Dashed arrows indicate biochemically uncharacterized reactions. Crosses indicate reactions that are not possible (red) or thought impossible on the basis of enzyme characterization experiments or the feeding study result (orange). *GGK and GGPK have not been shown to be active on (partially) saturated isoprenoids. ** GGGPS can utilize phytyl-PP as a prenyl donor, but this has not been demonstrated for DGGGPS. *** Due to the substrate promiscuity of GGR (toward headgroups) and headgroup diversification enzymes (toward the lipid backbone and radyl tails), isoprenyl saturation and headgroup modification could occur in parallel

Many archaea and some bacteria synthesize macrocylic glycerol dialkyl glycerol tetraether lipids (GDGTs) which resemble two tail-to-tail dimerized dialkyl glycerol diether lipids (DGDs) (de Rosa et al. 1977; Damsté et al. 2007; Schouten et al. 2007; Sinninghe Damsté et al. 2014; Naafs et al. 2018) (Fig. 14). In some archaea, such as *S. acidocaldarius*, GDGTs are the main constituent of the cell membrane forming a predominantly monolayer membrane (Rohr et al. 2010; Jensen et al. 2015b; Quehenberger et al. 2020; Tourte et al. 2020). In Crenarchaeota and Euryarchaeota, GDGTs can contain up to eight cyclopentane rings in the dialkyl moieties of the caldarchaeol lipid core, while in Thaumarchaeota, up to four cyclopentane rings can be found, with an extra cyclohexane ring to form the crenarchaeol lipid core unique to this clade (Schouten et al. 2000; Hopmans et al. 2000; Sinninghe Damsté et al. 2002). GDGTs can be found modified in various other ways such as the addition of methyl groups, bridging of the alkyl moieties, or the use of a butane- or pentane-triol backbone instead of glycerol, resulting in a diverse spectrum of GDGT derivatives (Morii et al. 1998; Knappy et al. 2015; Becker et al. 2016). The proportion of GDGTs in the membrane is subject to homeoviscous adaptation and tends to increase at higher cultivation temperatures (Sinensky 1974; Lai et al. 2008). Additionally, the degree of cyclization in GDGTs also increases at higher temperatures and this has motivated the use of GDGT-containing fossil remains as historical temperature proxies (Uda et al. 2001, 2004; Schouten et al. 2002; Shimada et al. 2008). On the other hand, the relation of the degree of GDGT pentacyclization and the pH is still unclear (Schouten et al. 2007; Shimada et al. 2008; Boyd et al. 2013). It has been suggested that the introduction of pentacyclic rings allows for more dense lipid packing, which aids the growth of hyperthermophiles by stabilizing the membrane structure at high temperatures likely decreases proton permeability (Gabriel and Lee Gau Chong 2000; Nicolas 2005; Pineda De Castro et al. 2016). In contrast, the hexane ring in crenarchaeol is thought to serve the opposite function, destabilizing lipid packing to form a more fluid membrane, for instance to allow for growth of Thaumarchaeota at lower temperatures (Sinninghe Damsté et al. 2002).

The exact biosynthetic route of GDGTs has for long remained elusive since the discovery of their structure more than 4 decades ago (Langworthy 1977). Several studies have touched upon this topic with varying hypotheses and mixed results. Pulse-labeling experiments revealed that *Thermoplasma* cells initially incorporate [^14^C]MVA in the archaeol (DGD) core lipid with the signal in the caldarchaeol (GDGT) core lipid increasing after the signal in DGD reached its maximum intensity (Langworthy 1982). This suggested that DGDs are a direct precursor to GDGT. Later experiments also used pulse-chase labeling techniques and verified the aforementioned observations (Nemoto et al. 2003b). Another study showed that terbinafine, a squalene-epoxidase inhibitor, inhibits GDGT synthesis resulting in DGD accumulation in *T. acidophilum* and that particular GDGT-exclusive headgroups are attached only after synthesis of GDGT from DGD (Petranyi et al. 1984; Ryder 1985; Kon et al. 2002; Nemoto et al. 2003b). On the other hand, a feeding experiment with [^14^C] radiolabeled saturated DGD or GDGT core lipids for incorporation in the membranes of *M. hungatei* did not show any interconversion of radioactivity from DGD into GDGT (Poulter et al. 1988). It appeared that double bonds in geranylgeraniol are important for incorporation of a [^3^H] radiolabel in both DGD and GDGT lipids, providing a possible explanation as to why the radioactivity of saturated DGD is not converted into the GDGT fraction (Poulter et al. 1988) (Fig. 14). A feeding study employing [^32^P]PO_4_^3−^ with *M. hungatei* showed that the label is readily incorporated in DGD phospholipids whereupon incorporation into GDGT phospholipids started later and was slower (Nishihara et al. 1989). This suggests that GDGT phospholipids have a larger precursor pool than DGD phospholipids, supporting the notion that DGDs are potentially precursors of GDGTs in *M. hungatei*.

In recent years, several of the key enzymes involved in formation of GDGTs have been identified. This concerns two GDGT (cyclopentane) ring synthases (GrsA and GrsB) and the tetraether (GDGT) synthase (Tes) from *S. acidocaldarius* and methanogens *(Methanococcus aeolicus* and *M. acetivorans),* respectively (Zeng et al. 2019, 2022)*.* These enzymes are part of the radical SAM superfamily of enzymes which use a [4Fe-4S]^+^ cluster to cleave S-adenosylmethionine to typically generate a 5’-deoxyadenosyl radical. This radical can be used to perform a large variety of reactions involving relatively unreactive substrates, including the formation of carbon–carbon bonds. This function finds use in enzymes that are involved in various critical cellular processes, such as co-factor synthesis, DNA methylation, enzyme activation and other protein post-translational modifications, including lipid-headgroup and lipid-tail modifications (Frey et al. 2008; Zeng et al. 2018, 2019, 2022) (Fig. 14).

Tes being a radical SAM enzyme strongly reinforced the notion that GDGTs are formed from the dimerization of diether lipids. However, the question whether the terminal double bonds of the diether substrate are required for catalysis remains unanswered. In 2000, Eguchi et al. presented dissimilar ^1^H NMR spectra between multiple-deuterated DGD lipids obtained by lipid extraction and purification from D_9_-mevalonolactone fed cultures of *Haloarcula japonica*, *M. thermoautotrophicus* and *M. jannaschii* (Eguchi et al. 1998, 2000). The dissimilar deuterium labeling was attributed to an unusual double-bond migration and linked with the presence or absence of GDGTs in the lipidome. It was suggested that this unusual double-bond migration leads to the formation of a terminal methylene group; enabling the synthesis of GDGTs from unsaturated diether lipids with a reductive coupling by an oxidoreductase at the step of double-bond saturation (Eguchi et al. 2000). This hypothesis was later retracted (Eguchi et al. 2003). Moreover, structural analysis of the GGR active site strongly suggests that GGR is not able to perform such proposed reductive coupling as the product would be unable to leave the active site of the enzyme the moment the macrocycle would be formed. Furthermore, it was shown that the terminal carbon atom retained both deuterium atoms upon formation of GDGT in *M. jannaschii* (Eguchi et al. 2000). This observation is highly similar to a study on diabolic acid synthesis in *Butyrivibrio fibrisolvens* fed with C-16 ^2^H_3_ palmitic acid where the resulting diabolic acid was shown to retain all 6 deuterium atoms (Fitz and Arigoni 1992). This excludes the formation of higher oxidation states of the terminal carbon atom during carbon–carbon-bond formation in both cases and is consistent with a radical mechanism. The latter may be similar to that of GDGT formation, suggesting that terminal double bonds in the diether substrate are not required for catalysis.

A later study involved the feeding of cultures of *M. thermoautotrophicus* with DGGGP di-methyl esters with various saturation patterns (Eguchi et al. 2003). Fully unsaturated DGGGP di-methyl ester was found to be incorporated into GDGTs and diethers, while a feed with a DGGGP di-methyl ester with the terminal bonds saturated was incorporated in diethers only (Fig. 14). If upstream processes are not taken into account, these data suggest that DGGGP or an equivalent molecule is the substrate for the GDGT synthase and that the terminal double bond is essential for GDGT synthase activity in *M. thermoautotrophicus.* Studies with membrane fractions of *M. thermoautotrophicus* suggest that CarS prefers DGGGP without saturations (Morii et al. 2000), which implies that lipids are typically saturated *after* headgroup activation. Thus, the DGGGP di-methyl ester with the saturated terminal double bonds might not be accepted by CarS and therefore not further processed into GDGTs (Fig. 14). Counterintuitively, the substrate specificity of CarS could prevent the incorporation of this molecule into GDGTs if the GDGT synthase is specific for saturated substrates or substrates with a particular headgroup. It should be noted that the proposed specificity of CarS for unsaturated substrates (Morii et al. 2000) needs to be verified with the purified enzyme in assays that allow for a better kinetic resolution using both saturated and unsaturated substrates.

Current insights suggest that the mechanism to form GDGTs is similar to the formation of diabolic acid that also involves a radical mechanism (Fitz and Arigoni 1992). This mechanism is advantageous as the terminal isoprenyl moiety might not need to remain unsaturated for GDGT synthesis. Considering GGRs substrate promiscuity, this would be much simpler to regulate and would imply that most GDGT precursors (GGPP, GGGP and DGGGP) remain unsaturated until the headgroup of DGGGP has been activated by CarS, forming CMP-DGGGP (CDP-archaeol). GGR could then reduce the double bonds of various diether lipids formed after headgroup activation by CarS (Nishimura and Eguchi 2006), which then would allow GDGT formation.

Reports on the directionality of the progressive nature of double-bond saturation are conflicting (Kung et al. 2014; Meadows et al. 2018). However, progressive saturation starting at the proximal double bonds toward the terminal double bonds would be an effective mechanism to aid in preventing the formation of unsaturated GDGTs, regardless of whether the GDGT synthase requires saturated or unsaturated terminal isoprenyl moieties. If the GDGT synthase indeed requires the terminal isoprenyl moiety to be saturated, and recognizes a headgroup, this mechanism would avoid the potential GDGT saturation problem and explain the observations of the feeding studies published so far. In the less likely case that the GDGT synthase requires unsaturated terminal bonds, it has to recognize the state of the other bonds in addition to the terminal double bond to prevent the formation of unsaturated GDGTs. Alternatively, the formation of unsaturated GDGTs would require the existence of an unidentified reductase specific for tetraethers; as from the active site geometry, it is unlikely that GDGTs are substrates for GGR. Overall, it seems much more likely that the GDGT synthase is specific toward saturated double bonds and employs a radical reaction mechanism similar to that of diabolic acid formation (Fitz and Arigoni 1992).

Intuitively, Tes was expected to be an integral membrane protein as the substrate is likely embedded in the membrane. However, the enzyme does not contain any transmembrane helices or domains (TMHMM V2, (Krogh et al. 2001)) (Zeng et al. 2022). Also, the recently identified GDGT cyclopentane ring synthases are not integral membrane proteins (Zeng et al. 2019), whereas they are thought to catalyze a reaction in the lipid tails of GDGTs which are buried in the membrane. Interestingly, GGR enzymes are also not integral membrane proteins either, but can still be found associated with the membrane (Nishimura and Eguchi 2006; Murakami et al. 2007; Sato et al. 2008; Meadows et al. 2018). Therefore, the question arises on how these enzymes reach their substrate (or vice versa). A possible solution can be found in the mechanism of Psd and several other enzymes; these enzymes bind to the polar surface of the membrane and provide a diffusion pathway to ‘extract’ the lipid from the membrane bilayer into the active site (Miller et al. 2008; Burke and Dennis 2009). A possibly similar but remarkable mechanism is the way by which the bacterial cyclopropane fatty acid synthase is proposed to add a methylene group, derived from S-adenosylmethionine, across the carbon–carbon double bond of unsaturated fatty acid radyl groups. This enzyme has been proposed to bind to the membrane surface, lifting or flipping the acyl region of the unsaturated fatty acid out of the hydrophobic interior of the phospholipid bilayer into the catalytic site (Grogan and Cronan 1997; Hari et al. 2018).

As discussed above, most studies suggest a mechanism involving the tail-to-tail dimerization of diethers to form tetraethers (Fig. 14), which is also the mechanism proposed for the recently discovered tetraether synthase Tes (Zeng et al. 2022). This is further substantiated by the observation that in some organisms, tetraether lipids are found where only one of the carbon–carbon bonds appears to have been formed [referred to as glycerol trialkyl glycerol tetraethers (GTGTs)] (Gulik et al. 1988; Hopmans et al. 2000; De La Torre et al. 2008; Elling et al. 2014) which is considered an intermediate in the tail-to-tail condensation process (Koga and Morii 2007; Zeng et al. 2022). An alternative GDGT biosynthetic pathway has been proposed in which IPPS is able to accept IPP intermediates containing pentacyclic rings, suggesting that the C_20_ isoprenoid-pyrophosphates dimerize to form C_40_ di-pyrophosphate isoprenoids with and without rings (Villanueva et al. 2014). These precursors would then be ether-bonded, at both ends, to G1P by GGGPS and DGGGPS forming GDGTs. Obviously, this would require an unprecedented level of functional plasticity of the phospholipid biosynthetic enzymes, or alternatively, involve so far undiscovered enzymes. Notably, GGPPS, GGGPS and DGGGPS must be able to accept C_>20_-isoprenoid substrates that can also contain rings. In this respect, the hydrophobic substrate-binding grooves or tunnels of AfGGGPS and MjDGGGPS do not appear deep enough to accept C_40_-isoprenoids and are unlikely to be wide enough to accommodate pentacyclic rings (Payandeh et al. 2006; Ren et al. 2020) (Figs. 4b and 6b) despite the fact that *A. fulgidus* and *M. jannaschii* are both GDGT-synthesizing organisms (Nichols et al. 2004; Lai et al. 2008). Finally, the proposed pathway suggests that isoprenyl chain saturation occurs after addition of the second glycerol moiety. The GGR substrate-binding cavity is split in two pockets (Xu et al. 2010; Sasaki et al. 2011) (Fig. 13c). For an isoprenyl moiety to bind in the active site, the isoprenyl tails of a typical diether isoprenoid lipid substrate are wedged apart. Due to the macrocyclic structure of GDGTs, the isoprenyl chains can no longer be wedged apart, and therefore, it is difficult to envisage how an unsaturated GDGT isoprenyl moiety would reach the active site of GGR.

Biphytane diols containing ring moieties have been detected in environmental samples and glycerol dialkanol diethers in both environmental samples and archaeal cultures of methanogens (Schouten et al. 1998; Liu et al. 2012; Knappy and Keely 2012). Biphytane diols could be produced from GGPP if the GDGT synthase and GrsA/GrsB accept Phytanyl-PP and bi-phytanyl-PP, respectively; as GGRs, at least in vitro, can accept GGPP (See GGR section). The proposition of glycerol dialkanols as precursors of GDGTs would be mechanistically quite challenging as both glycerol dialkanol tails would need to attach to the same glycerol moiety, and in the correct position, to form the GDGT macrocycle. Reported glycerol dialkanol diethers are fully saturated and lack a phosphate headgroup (Liu et al. 2012; Knappy and Keely 2012). As described above, known prenyltransferases require the prenyl donor to have a pyrophosphate “head-” group and the proximal isoprenyl moiety to be unsaturated. Thus, the hydroxyl groups characteristic of glycerol dialkanols would need to be phosphorylated by enzymes related to geranylgeraniol kinase (GGK) and GGP kinase (GGPK) that would need to accept longer isoprenoid substrates (Ohnuma et al. 1996c). Additionally, an unidentified enzyme would be required to create the macrocycle by the attachment of the second glycerol moiety. Moreover, lipids are synthesized with a phosphate headgroup originating from G1P, which plays an important role in substrate binding by both GGGPS and DGGGPS. This phosphate headgroup is either retained for phospholipid synthesis, or is likely eliminated after the diether phospholipid core has formed for—or during the synthesis of other lipid types such as glycolipids (Morii et al. 2007; Zeng et al. 2018). As such, a more feasible explanation would be that these molecules are breakdown products of GDGTs. The intermediates of the proposed alternative biosynthetic pathway would be expected to be widely present among GDGT-synthesizing archaea. So far, reports detecting these suggested intermediates in lipidomes are scarce and evidence for the unprecedented enzyme functional plasticity is lacking. Thus, tail-to-tail dimerization of diether lipids by GDGT synthase remains the most likely mechanism for tetraether lipid formation.

### GDGT cyclopentane and cyclohexane ring formation

As mentioned before, GDGTs can carry up to eight cyclopentane rings and/or one cyclohexane ring. Ring formation is an important adaptation to environmental conditions, such as changes in growth temperature. Two GDGT ring synthases, GrsA and GrsB, have been identified to be essential for GDGT ring formation in *S. acidocaldarius* (Zeng et al. 2019). Both proteins are radical SAM proteins, indicating that GDGT cyclization occurs through a free radical mechanism. The genes encoding both enzymes were deleted in *S. acidocaldarius*, and neither gene appeared essential. However, the deletions allow a functional assignment of their function as it resulted in a dramatic reduction of the number of rings in GDGT. GrsA introduces rings specifically at the C-7 position of the core GDGT lipid, while GrsB cyclizes at the C-3 position. The ring at the C-7 position is introduced prior to the ring at the C-3 position. Cyclization occurs after the formation of DGGGP and after the condensation of two diether lipids by Tes to form GDGT. It is, however, unclear whether the GDGT substrate must be unsaturated for cyclization and whether different phospholipid and hexose head groups influence ring formation. Since the cyclization patterns are differentially controlled by the two enzymes, this provides a potential mechanism to adjust the composition of the membrane in response to environmental factors. A GDGT structure that appears to be restricted to Thaumarchaeota species, named crenarchaeol, contains 4 cyclopentane rings at C-7 and a unique cyclohexane ring at C-11. Likely, Thaumarchaeota harbor a GrsA homolog as well as another Grs responsible for generating the C-11 cyclohexane ring. However, this enzyme has so far not been identified.

### Formation of the calditol polar head group

Calditol is a unique cyclopentyl head group that occurs through an unusual ring contraction of a glucose molecule ether-linked to the glycerol backbone of GDGTs. This polar headgroup is found in a subset of thermoacidophilic archaea of the Sulfolobales order within the Crenarchaeota phylum. Calditol formation *S. acidocaldarius* depends on a radical SAM protein termed calditol synthase (Cds) (Zeng et al. 2018). The enzyme is found not only in Crenarchaeota, but also in other phyla including the Korarchaeota and Marsarchaeota, while its presence in metagenomes is mostly linked to acidic ecosystems. Deletion of calditol synthesis renders *S. acidocaldarius* sensitive to extremely low pH, indicating that calditol plays a critical role in protecting archaeal cells from acidic stress (Zeng et al. 2018). A mechanism of calditol formation has been proposed where a monohexose lipid—which is first synthesized by a glucosyl-transferase—would be the substrate for the calditol synthase. Following the radical reaction, which leads to the glycosyl ring contraction, a second glucosyl-transferase would subsequently add a hexose group to calditol to generate a monohexose calditol head group.

## Concluding remarks

The glycerophospholipid core biosynthesis pathway is fairly well characterized in both Archaea and Bacteria, but a particularly interesting group of organisms remains. In recent years, the new “Asgard” superphylum of archaea has been described. Members of this superphylum include the Thor-, Odin-, Heimdall- and Lokiarchaea, and are of particular interest as these archaea are the most closely related members of the Archaea to Eukaryotes (Zaremba-Niedzwiedzka et al. 2017; Spang et al. 2018; Fournier and Poole 2018). The first Lokiarchaeal metagenome was found to not contain a complete archaeal phospholipid biosynthesis pathway, lacking G1P dehydrogenase (G1PDH) (Spang et al. 2015; Villanueva et al. 2017). Surprisingly, Lokiarchaea were found to possess homologs to PlsC and only PlsY of the PlsXY pathway which are both typically only found in Bacteria, leading to the proposition that Lokiarchaea might synthesize archaeal phospholipids with a G3P backbone or chimeric phospholipids containing both archaeal and bacterial radyl groups. Later metagenome sequencing studies resulted in more Lokiarchaeal genomes which revealed the presence of a G1PDH, suggesting that Lokiarchaea are capable of synthesizing complete archetypical archaeal phospholipids (Manoharan et al. 2019). Indeed, isoprenoid-based lipids have been found in the only Lokiarchaeote that could be cultivated so far (Imachi et al. 2020). However, the function of the PlsY and PlsC homologs remains to be elucidated. For this reason, Lokiarchaea are an interesting group of organisms to study for the structural basis for archaeal versus bacterial phospholipid biosynthesis and the second lipid-divide that must have occurred during the early evolution of eukaryotes.

The synthesis of the glycerophospholipid cores in Archaea and Bacteria occurs along similar lines while employing different molecular mechanisms (Fig. 1); for example, the utilization of pyrophosphate groups for the formation of the ether bonds in Archaea compared to the transesterification of acyl-ACP to glycerol in Bacteria. The reactions involving CarS and CAPT enzymes for headgroup activation and diversification are saliently similar between Archaea and Bacteria (Fig. 1 and 8). However, despite the common presence of CAPT enzymes in both pathways, only relatively little structural and biochemical information has been reported. For instance, Ags is thought to attach G3P as a headgroup, but this enzyme has not been characterized (Caforio et al. 2015). In Bacteria, G3P is utilized both for the phospholipid backbone and the phosphatidylglycerol polar headgroup. Archaea utilize G1P for the phospholipid backbones, but as not all archaea seem to possess typical G3P synthesis-related enzymes, it remains to be determined whether the lipid-divide extends to the headgroup of AG and which chiral form of glycerol phosphate is used by Ags (Villanueva et al. 2017).

The substrate specificities of CarS and GGR are two key points that have insufficiently been addressed. While a particular group of GGRs seems to use ferredoxin as a biological reducing agent, the biological reducing agent of many GGRs is still not known. In vitro studies using sodium dithionite as an artificial reducing agent suggest that GGR is a rather promiscuous enzyme as it accepts free isoprenols and synthetic isoprenoid esters, GGPP, GGGP, DGGGP and perhaps even CDP-lipids and mature phospholipids with diverse polar headgroups. Experiments with crude lysates of *M. thermoautotrophicus* suggest a specificity of MtCarS for unsaturated phospholipids, suggesting that phospholipid saturation by GGR occurs after headgroup activation by CarS. However, this needs to be further examined with the purified enzyme in kinetic assays. Does the in vitro activity of GGR demonstrate the true substrate specificity of GGR? Or is the biosynthetic pathway more organized in space where GGR acts only on CDP-archaeol and downstream products? When it comes to substrate specificity for the glycerol backbone, GGGPS, and likely DGGGPS, are specific toward substrates with the G1P stereochemistry. On the basis of the CarS crystal structure, CarS is expected to be specific for G1P-based substrates, as well. However, this has not been tested in vitro with a purified enzyme. A thorough biochemical characterization of GGR and CarS, and protein–protein interactions of lipid biosynthesis enzymes, are of prime interest to further our knowledge on lipid biosynthesis in Archaea.

Recently, two GDGT ring synthases (GrsA and GrsB (Zeng et al. 2019)) and the tetraether (GDGT) synthase were identified (Zeng et al. 2022). However, the enzymes have not been biochemically characterized. For instance, are GrsA and GrsB only active on tetraethers and not on diethers or substrates with a single radyl group? While the GDGT synthase is expected to tail-to-tail couple diether lipids, its substrate specificity has not been studied either. A key question remains as to whether the GDGT synthase is active on saturated or unsaturated terminal isoprenyl groups. Also, it remains to be determined how substrate recognition is accomplished for all of these enzymes; as GrsA, GrsB and the GDGT synthase are not predicted to be integral membrane proteins, while their substrates with their sites of catalysis are thought to be buried in the membrane. Biochemical and structural characterization is key toward a better understanding of the biochemical and mechanistic basis of GDGT formation, and the formation of related lipids such as cyclic diethers and other tetraethers such as H-bridged lipids, crenarchaeol, bacterial bridged-GDGTs and potentially other lipids with unusual carbon structures.

## Abbreviations

AA: Archaetidic acid
aCL: Archaeal CL
ACP: Acyl carrier protein
AE: Archaetidylethanolamine
AG: Archaetidylglycerol
AGP: Archaetidylglycerol phosphate
Agp: Archaetidylglycerol phosphatase
Ags: Archaetidylglycerol synthase
AI: Archaetidylinositol
AIP: Archaetidylinositol phosphate
Aip: Archaetidylinositol phosphatase
Ais: Archaetidylinositol synthase
AMPD: Anhydromevalonate phosphate decarboxylase
ArtA: Archaeosortase A
AS: Archaetidylserine
Asd: Archaetidylserine decarboxylase
ASR: Ancestral structure reconstruction
Ass: Archaetidylserine synthase
bCL: Bacterial CL
BLAST: Basic local alignment search tool
BMD: Bisphosphomevalonate decarboxylase
CAPT: CDP-alcohol phosphatidyl transferase
CarS: CDP-archaeol synthase
CDP: Cytidine diphosphate
CdsA: CDP-DAG synthase
CL: Cardiolipin
Cls: Cardiolipin synthase
CMP: Cytidine monophosphate
CoA: Coenzyme A
COG: Cluster of orthologous genes
CoQ2: 4-Hydroxybenzoate polyprenyltransferase
CoX10: Protoheme IX farnesyltransferase
CTP: Cytidine triphosphate
DAG: Diacylglycerol
DGD: Dialkyl glycerol diether
DGGGP: Di-geranylgeranylglycerol phosphate
DGGGPS: Di-geranylgeranylglycerol phosphate synthase
DMAPP: Dimethylallyl pyrophosphate
DMD: Diphosphomevalonate kinase
EDTA: Ethylenediaminetetraacetic acid
FabA: 3-Hydroxydecanoyl-[acyl-carrier-protein] dehydratase
FabB: 3-Oxoacyl-[acyl-carrier-protein] synthase
FabI: Enoyl-[acyl-carrier-protein] reductase
FabZ: 3-Hydroxyacyl-[acyl-carrier-protein] dehydratase
FAD: Flavin adenine dinucleotide
FARM: First aspartate-rich motif
FAS: Fatty acid synthase
FPP: Farnesyl pyrophosphate
FPPS: Farnesyl pyrophosphate synthase
G1P: Glycerol-1-phosphate
G1PDH: G1P dehydrogenase
G3P: Glycerol-3-phosphate
GDGT: Glycerol dialkyl glycerol tetraether
GDGTS: GDGT synthase
GFPP: Geranylfarnesyl pyrophosphate
GFPPS: Geranylfarnesyl pyrophosphate synthase
GGGP: Geranylgeranylglycerol phosphate
GGGPS: Geranylgeranylglycerol phosphate synthase
GGK: Geranylgeraniol kinase
GG-OH: Geranylgeraniol
GGPK: Geranylgeranyl phosphate kinase
GGPP: Geranylgeranyl pyrophosphate
GGPPS: Geranylgeranyl pyrophosphate synthase
GGR: Geranylgeranyl reductase
GPP: Geranyl pyrophosphate
Gro-DACL: Glycerol-di-archaetidyl-CL
Gro-DPCL: Glycerol-di-phosphatidyl-CL
Grs: GDGT ring synthase
HepPPS: Heptaprenyl pyrophosphate synthase
HexPPS: Hexaprenyl pyrophosphate
I1P: (1L-*myo*-) Inositol-1-phosphate
IDI: Isopentenyl pyrophosphate: dimethylallyl pyrophosphate isomerase
IP: Isopentenyl phosphate
IPP: Isopentenyl pyrophosphate
IPPS: Isoprenyl pyrophosphate synthase
LBG: Lipid-binding groove
LC–MS: Liquid chromatography–mass spectrometry
LDAO: Lauryldimethylamine oxide
LPA: Lyso-phosphatidic acid
LUCA: Last universal common ancestor
M3K: Mevalonate-3 kinase
M3P5K: Mevalonate-3-phosphate-5 kinase
M5K: Mevalonate-5-kinase
MD: Molecular dynamics
MenA: 1,4-Dihydroxy-2-naphthoate octaprenyltransferase
MVA: Mevalonate
MVA-3,5-PP: Mevalonate-3,5-biphosphate
MVA-3-P: Mevalonate-3-phosphate
MVA-5-P: Mevalonate-5-phosphate
MVA-5-PP: Mevalonate-5-pyrophosphate
MVK: Mevalonate kinase
NADPH: Nicotinamide adenine dinucleotide phosphate
PA: Phosphatidic acid
PcrB: Heptaprenylglycerol phosphate synthase
PDB: Protein data bank
PE: Phosphatidylethanolamine
PG: Phosphatidylglycerol
PGP: Phosphatidylglycerol phosphate
Pgp: Phosphatidylglycerol phosphatase
Pgs: Phosphatidylglycerol synthase
PHBH: *p*-Hydroxy-benzoate hydroxylase
PI: Phosphatidylinositol
PIP: Phosphatidylinositol phosphate
Pip: Phosphatidylinositol phosphatase
Pis: Phosphatidylinositol synthase
PLD: Phospholipase D
PlsB: Glycerol-3-phosphate acyltransferase
PlsC: 1-Acyl-sn-glycerol-3-phosphate acyltransferase
PlsX: Phosphate acyl transferase
PlsY: Glycerol-3-phosphate acyltransferase
PMD: Phosphomevalonate decarboxylase
PMDh: Phosphomevalonate dehydratase
PMFPLD: *Streptomyces* Sp. PMF PLD
PMK: Phosphomevalonate kinase
PPi: Inorganic pyrophosphate
PS: Phosphatidylserine
Psd: Phosphatidylserine decarboxylase
Pss: Phosphatidylserine synthase
SARM: Second aspartate-rich-motif
SCD: Schnyder corneal dystrophy
tAMVA-5-P: *trans*-Anhydromevalonate-5-phosphate
TES: Tetraether synthase
TIM-barrel: Triose phosphate isomerase barrel
TM: Transmembrane
UbiA: 4-Hydroxybenzoate octaprenyltransferase

## Footnotes

1 In this work “archaeol” does not specifically refer to di-phytanyl-glycerol or di-sesterterpanyl-glycerol but rather as a general term for the archaeal diether glycerol core lipid regardless of its saturation state.

2
*E. coli* PssA is atypical as it is a member of the phospholipase D family containing the prototypical HKD motif but capable of forming the same phosphodiester bond as members of the CAPT family

3 This work only discusses 1L-*myo*-inositol. From this point onwards “1L-*myo*-“ is omitted for reading clarity.
